# *Tripterygium hypoglauc*um (Lévl.) Hutch and Its Main Bioactive Components: Recent Advances in Pharmacological Activity, Pharmacokinetics and Potential Toxicity

**DOI:** 10.3389/fphar.2021.715359

**Published:** 2021-11-23

**Authors:** Junqi Zhao, Fangling Zhang, Xiaolin Xiao, Zhao Wu, Qichao Hu, Yinxiao Jiang, Wenwen Zhang, Shizhang Wei, Xiao Ma, Xiaomei Zhang

**Affiliations:** ^1^ State Key Laboratory of Southwestern Chinese Medicine Resources, School of Pharmacy, Chengdu University of Traditional Chinese Medicine, Chengdu, China; ^2^ Hospital of Chengdu University of Traditional Chinese Medicine, School of Clinical Medicine, Chengdu University of Traditional Chinese Medicine, Chengdu, China; ^3^ Institute of Medicinal Chemistry of Chinese Medicine, Chongqing Academy of Chinese Materia Medica, Chongqing, China

**Keywords:** *Tripterygium hypoglaucum* (Lévl.) Hutch., phytochemistry, pharmacology, pharmacokinetics, toxicity

## Abstract

*Tripterygium hypoglaucum* (Lévl.) Hutch (THH) is believed to play an important role in health care and disease treatment according to traditional Chinese medicine. Moreover, it is also the representative of medicine with both significant efficacy and potential toxicity. This characteristic causes THH hard for embracing and fearing. In order to verify its prospect for clinic, a wide variety of studies were carried out in the most recent years. However, there has not been any review about THH yet. Therefore, this review summarized its characteristic of components, pharmacological effect, pharmacokinetics and toxicity to comprehensively shed light on the potential clinical application. More than 120 secondary metabolites including terpenoids, alkaloids, glycosides, sugars, organic acids, oleanolic acid, polysaccharides and other components were found in THH based on phytochemical research. All these components might be the pharmacological bases for immunosuppression, anti-inflammatory and anti-tumour effect. In addition, recent studies found that THH and its bioactive compounds also demonstrated remarkable effect on obesity, insulin resistance, fertility and infection of virus. The main mechanism seemed to be closely related to regulation the balance of immune, inflammation, apoptosis and so on in various disease. Furthermore, the study of pharmacokinetics revealed quick elimination of the main component triptolide. The feature of celastrol was also investigated by several models. Finally, the side effect of THH was thought to be the key for its limitation in clinical application. A series of reports indicated that multiple organs or systems including liver, kidney and genital system were involved in the toxicity. Its potential serious problem in liver was paid specific attention in recent years. In summary, considering the significant effect and potential toxicity of THH as well as its components, the combined medication to inhibit the toxicity, maintain effect might be a promising method for clinical conversion. Modern advanced technology such as structure optimization might be another way to reach the efficacy and safety. Thus, THH is still a crucial plant which remains for further investigation.

## Introduction

Effect and toxicity are two important aspects of drugs. Among the compounds obtained from plants or natural sources, agents with remarkable efficacy and potential toxicity have attracted much attention in recent years. It is well-known that some traditional Chinese medicine is highly toxic such as arsenic, *Aconitum carmichaelii* Debx. and *Tripterygium wilfordii* Hook F. (TwHF). Indeed, long-term consumption of arsenic can lead to arsenic accumulation in vital organs with diabetes, atherosclerosis, hypertension, ischemic heart disease, and hepatotoxicity (Khairul et al., 2017). However, arsenic trioxide (As_2_O_3_) has recently shown significant efficacy in patients with acute promyelocytic leukemia (APL) and many other malignancies, such as adult T-cell leukemia/lymphoma (ATL) and NPM1 mutant acute myeloid leukemia (AML) (Kchour et al., 2009; Mathews et al., 2011; El Hajj et al., 2015). In addition, fatal ventricular tachycardia and asystole may occur if aconite poisoning is severe (Chan, 2012). The processed *Aconitum carmichaelii* Debx. plays an important role as cardiotonic, analgesic and anti-inflammatory in traditional medicine (Chan, 2011). Moreover, TwHF is frequently reported exhibiting multiple toxicity, including reproductive toxicity, renal cytotoxicity, and hepatotoxicity (Li X.-J. et al., 2014). However, due to its significant anti-inflammatory and immunosuppressive effects, TwHF preparations have been widely used for the treatment of autoimmune disorders and inflammatory diseases, such as rheumatoid arthritis (RA) (Luo et al., 2019; Xu et al., 2019). It is worth noting that multiple organ toxicity in TwHF can be resolved after dose adjustment (Zhang et al., 2021a). Therefore, the agents with both remarkable effect and potential toxicity should be paid specific focus.

**FIGURE 1:**
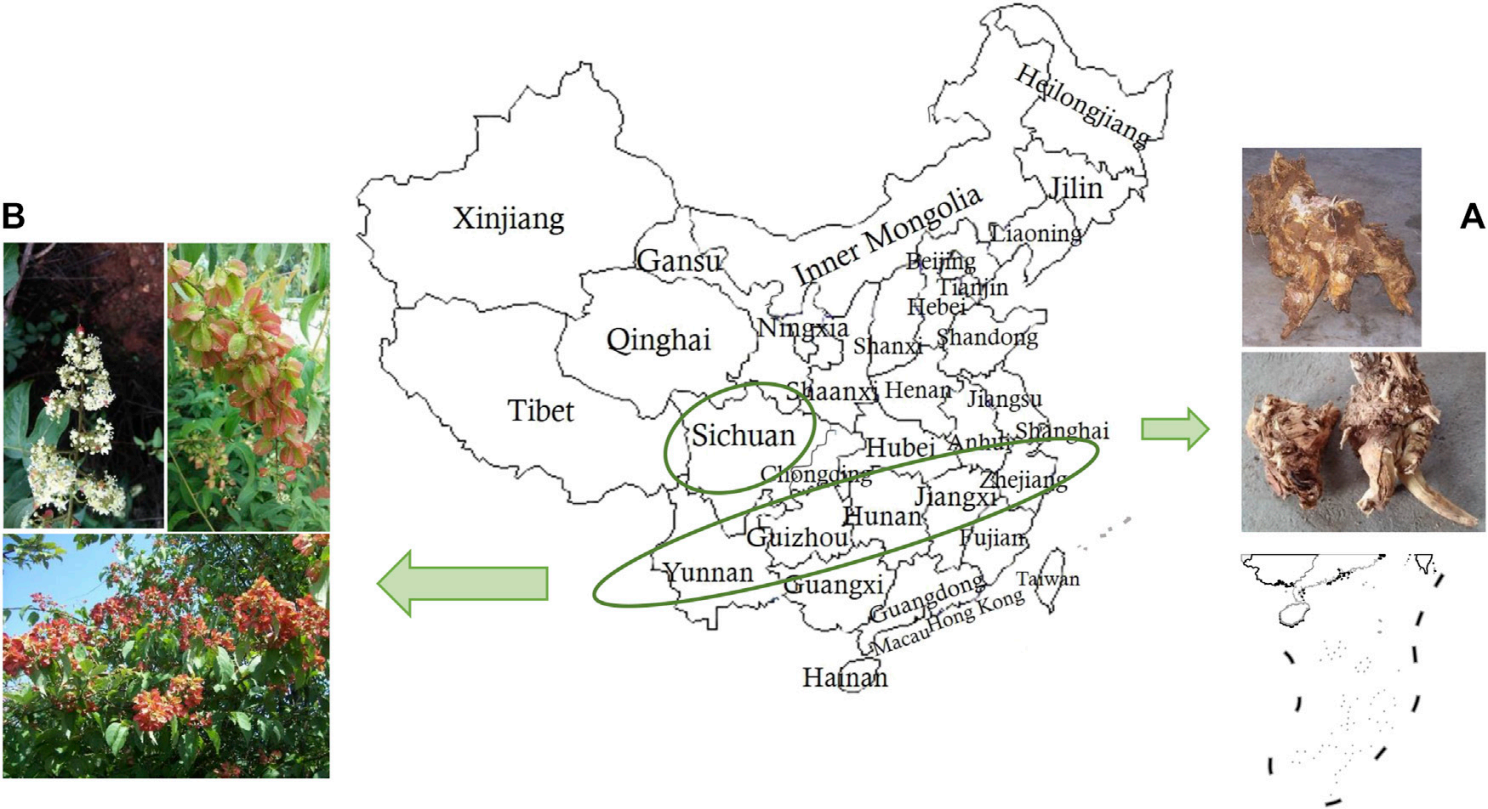
Climatic and ecological adaptability distribution of THH in China. **(A)** Dried root bark of THH, **(B)** Aerial view of THH.

*Tripterygium hypoglau*cum (Lévl.) Hutch. (THH), a plant distributed in southwest regions of China and along the south bank of the Yangtze River, has similar pharmacological effects with fewer side effects and lower toxicity than TwHF in the clinic (Li, 2006; Brinker et al., 2007; Ma et al., 2008) (Figure 1). Its main chemical components include alkaloids, diterpenoids and triterpenoids. Its root bark demonstrates excellent medicinal properties and has been widely used in folk medicine in China (Li, 2006). Numerous studies have found that terpenoids isolated from THH possess various pharmacological activities including anti-tumour, immunosuppressive and anti-inflammatory effects (Liu, 2011; Lü et al., 2015). Studies have shown that celastrol, a triterpenoid discovered from THH, relieves insulin resistance, reduces body weight, and suppresses polycystic kidney disease (Booij et al., 2020). Despite significant efficacy, the clinical application of THH has a narrow therapeutic window due to severe toxicity, thereby limiting its widespread application (Dudics et al., 2018; Yuan et al., 2019a; Wang et al., 2019).

Identification of the active chemical ingredients and pharmacological effects of THH is the primary goal of most THH studies, and this information is useful for the development of efficient and low-toxicity modern traditional Chinese medicines. The current research review on THH is limited, and sufficient attention has not been given to the relationship between compounds, pharmacological activities and toxicity. The chemical compositions and pharmacological mechanisms and potential toxicity of THH were studied to provide a scientific basis for the development of a safe medication with better clinical utility. To further clarify the chemical composition, mechanism of action, pharmacological effects and toxicity characteristics of THH, we conducted the following review. We also summarized the pharmacokinetics study of THH in animals.

## Methodology

Database searches using PubMed, China National Knowledge Internet (CNKI) and Wanfang database were conducted until December 2020. *Tripterygium hypoglaucu*m (Lévl.) Hutch., *Tripterygium*, triptolide, celastrol and kunmingshanhaitang were searched as key words in the databases mentioned above. All of databases were retrieved twice. Experimental articles related to THH and its active components were included, and some irrelevant literature was excluded through reading abstracts or the full text. In almost all cases, the original articles were obtained and the relevant data was extracted.

### Phytochemical Constituents

**FIGURE 2:**
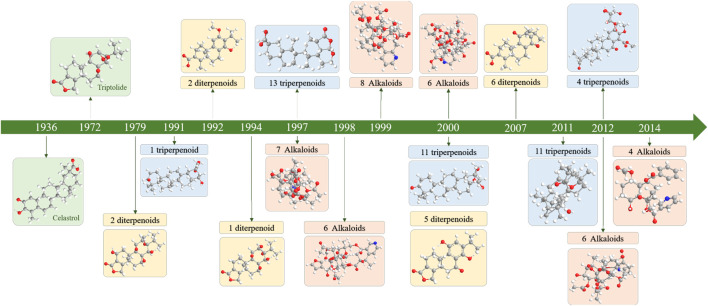
Timeline of the isolation and identification of main chemical components from the genus *Tripterygium*.

**FIGURE 3:**
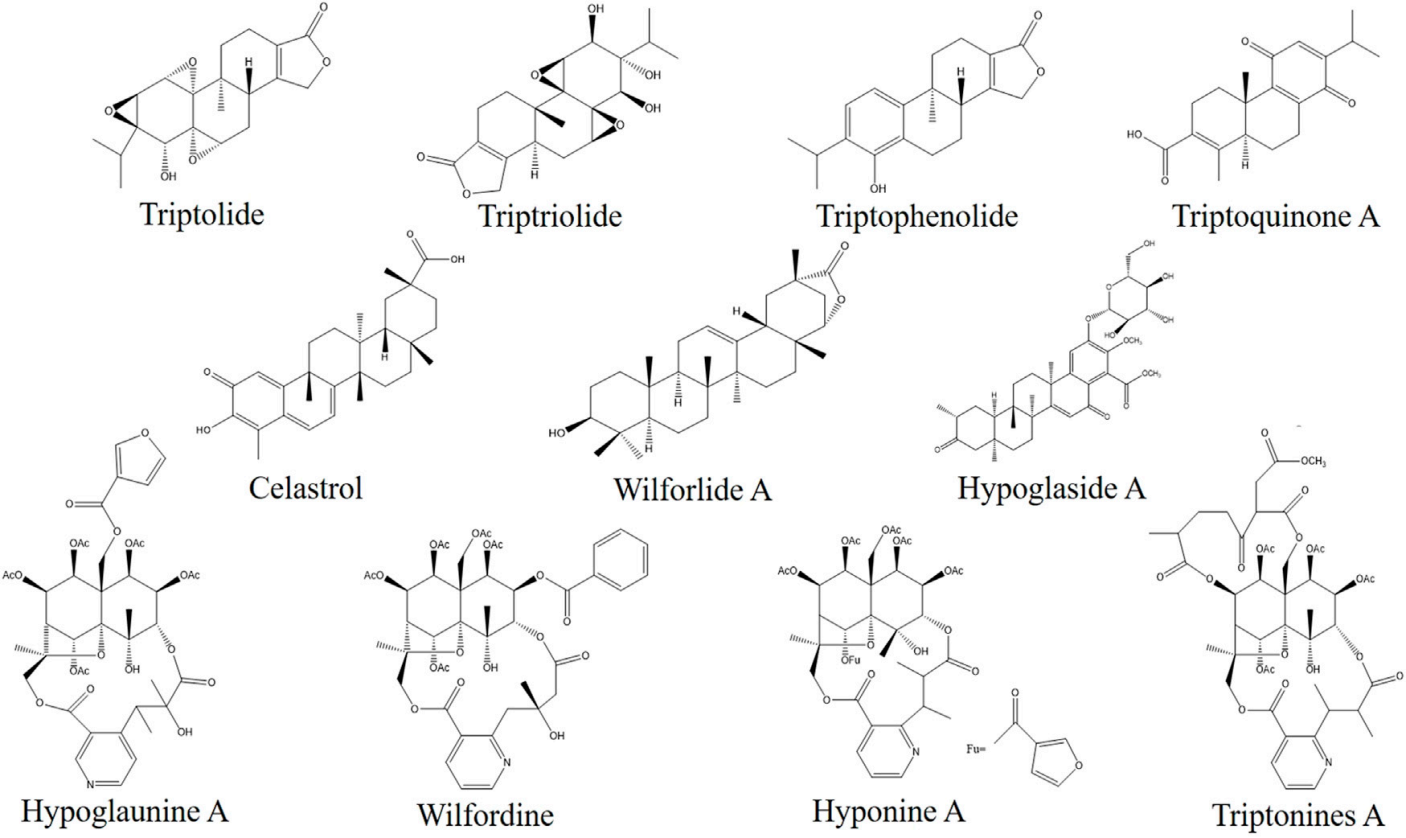
Chemical structures of the existing potential quality control markers in THH.

Since the 1960s, scholars have conducted in-depth research on TwHF through nearly half a century of chemical composition research on the genus *Tripterygium* (Figure 2). This plant genus contains more than 100 chemical components such as terpenoids (diterpenes, triterpenes, sesquiterpenes), alkaloids, glycosides, sugars, organic acids, euonymol, euonymus alkaloids, polysaccharides, β-sitosterol and other chemical components. Its characteristic active ingredients include diterpenoids such as triptolide, triptophenolide, triptriolide, triptoquinone A, and triterpenoids, such as celastrol and wilforlide A (Xie et al., 2015) (Figure 3).

In the earliest study (1972) of the chemical composition of THH, triptolide, triptolide diol, and triptolide ketone were isolated and identified from the ethanol-ethyl acetate extract of TwHF, a congeneric plant produced in China (Kupchan et al., 1972). In 1979, domestic studies in China first isolated trace amounts of two diterpene lactone chemical constituents, hypolide and tripterolide, from the root bark of TwHF. As a common chemical component of the three species of this genus, hypolides were isolated not only from THH but also from other species of the same genus, and it was hypothesized that hypolides might represent one of the precursors of biosynthesis in this genus (Wu and Sun, 1979).

A study reported that three rosin diterpenes, namely hypoglic acid, triptonoterpene methyl ether and triptonoterpenol could be separated from the ethyl acetate soluble portion of the root ethanol extract by silica gel column chromatography (Wang et al., 2012). An additional three ursolane triterpenes, namely hypoglaulide, triptotriterpenic acid C and regelin were obtained, and 3-acetoxy oleanolic acid was also obtained during the same separation process in 1992 (Zhang et al., 1992a). Ten crystals were obtained from the ethanol extract of the coarse powder of THH root: 3-oxofriedelan-29-oic acid methyl ester; β-sitosterol; 3-epik atonic acid methyl ester; 3β, 22α-dihydroxy-Δ12-oleanen-29-oic acid methyl ester; and 3β, 22α-dihydroxy-Δ12-ursen-30-oic acid methyl ester (Yi and Yang, 1993). In other studies, the chloroform extracts of THH roots were separated by column chromatography, and a total of 12 crystals were obtained. Of these, 8 compounds were identified in 1992, including tripteroquinone, triptolide A, triptolide B, tripterygidine, triptolide, 1-epicatechin and β-sitosterol (Zhang and Zhang, 1992). They also reported that three diterpenoid compounds, namely, triptoditerpenic acid, tritoditerpenic acid B and hypodiolide A, were obtained from the chloroform extracts of THH in the same year. In addition, other components, such as wilforgine and daucosterol, have been isolated from THH (Ding, 1991).

### Sesquiterpenoids

Sesquiterpenes be isolated from THH mostly combine with pyridine to form alkaloids. At present, only two sesquiterpenoid monomeric compounds have been isolated from THH. In 2000, a sesquiterpene compound was isolated from the methanol extract of the root bark of THH. The molecular formula was C_31_H_36_O_7_, and the structural formula was 1β-benzoyl-8α-cinnamoyl-4α, 5α-dihydroxy dihydroagarofuran (Fujita et al., 2000). In 2014, a sesquiterpene compound was isolated from the 95% ethanol extract of the root bark of THH. The molecular formula was C_31_H_36_O_8_ and the structural formula was: 1α-acetoxy-6β, 9β-dibenzoyloxy-4β-hydroxy-dihydroagarofuran. These compounds exhibited weak cytotoxicity against HeLa cells with an IC_50_ value of 30.2 µM (Liu Z.-Z. et al., 2014). Dihydro-β-agarofuran sesquiterpenes from THH are substrates of P-gp and potential modulators of MDR. These compounds exhibit no cytotoxicity in HepG2 and HepG2/Adr cells. Moreover, these compounds restored the sensitivity of HepG2/Adr cells to Dox and increased the accumulation of intracellular Dox (Yang et al., 2019).

### Diterpenoids

Diterpenoids are the most important components in THH, and triptolide exhibits the most remarkable activity. Regarding the structural types of diterpenoids, epoxy-type diterpenoids, randolactone-type diterpenoids, fulfolterpenoid diterpenoids, kaurane-type diterpenoids and abietane-type diterpenoids are primarily observed in THH.

One ranolactone-type diterpenoid, triptonolide, and two ranolterpene-type diterpenoids triptobenzene A and triptobenzene J were isolated from the methanol extract of THH (Duan et al., 1997). In addition, the following diterpenoids were isolated from the methanol extract of THH rhizomes in 2000: two ranolactone-type diterpenoids, triptobenzene K and triptophenolide; two fulfolterpenoid diterpenoids, triptobenzene D and triptobenzene L; and one abietane-type diterpenoid, quinone 21 (Fujita et al., 2000). In 2007, three fulfolterpenoid diterpenoids (triptobenzene H, triptonediol, and triptonoterpene) and three abietane-type diterpenoids (triptoquinone A, triptoquinone B, and triptoquinone H) were isolated from 95% ethanol extracts of THH (Zhang et al., 2007). In 2011, a new fulfolterpenoid diterpenoid named triptonoterpene methyl ether was isolated from a 95% ethanol extract of the root bark of THH (Liu et al., 2011). A randolactone-type diterpenoid, 11-O-β-D-glucopyranosyl neotriptophenolide was isolated from a 95% ethanol extract of THH (Li X, 2006). Four new diterpenoids, 2α, 16α-hydroxy-ent-kauran-19,20-olide, isopimara-8 (14), 15-diene-11β, 19-diol, isopimara-8 (14),15-diene-12α, 19-diol, and 3-oxo-14,15-dihydroxyabieta-8,11,13-trien-19-ol were isolated from THH. Three new terpenoid compounds, epoxyionone A, triptobenzene W and loliolide A, were isolated from the 95% EtOH extract of the twigs of THH (Zheng et al., 2020). A new diterpenoid, 19-O-β-D-glucopyranosyl-labda-8 (17), 14-dien-13-ol was isolated from the aerial parts of THH (Zhao et al., 2018) (Table 1).

**TABLE 1:** Diterpenoids isolated from *Tripterygium hypoglaucum.*

Serial number	Compound	Molecular formula	Plant part	References
1	Hypolide	C_20_H_24_O_3_	Root	Wu and Sun (1979)
2	Triptonoterpenol	C_21_H_30_O_4_	Root	Zhang et al. (1992b)
3	Hypoglic acid	C_20_H_26_O_4_	Root	Zhang et al. (1992b)
4	Triptonoterpene methyl ether	C_21_H_30_O_3_	Root	Zhang et al. (1992b)
5	Hypodiolide A	C_24_H_36_O_3_	Root	Zhang and Zhang (1992)
6	Triptoditerpenic acid B	C_21_H_28_O_3_	Root	Zhang and Zhang (1992)
7	Triptonoditerpenic acid	C_21_H_28_O_4_	Root	Zhang and Zhang (1992)
8	Triptoquinone H	C_20_H_26_O_3_	Root	Ding (1991)
9	Triptobenzene L	C_22_H_34_O_3_	Root	Ding (1991)
10	Triptolide	C_20_H_24_O_6_	Root	Duan et al. (1997)
11	Triptonolide	C_20_H_22_O_4_	Root	Duan et al. (1997)
12	Triptobenzene A	C_20_H_28_O_3_	Root	Duan et al. (1997)
13	Triptobenzene J	C_20_H_30_O_3_	Root	Duan et al. (1997)
14	Triptobenzene K	C_20_H_22_O_5_	Root	Duan et al. (1997)
15	Neotriptophenolide	C_21_H_26_O_4_	Root	Duan et al. (1997)
16	2α,16α-Hydroxy-ent-kauran-19,20-olide	C_20_H_30_O_4_	Stem	Li et al. (2015b)
17	Isopimara-8 (14), 15-diene-11*β*,19-diol	C_20_H_32_O_2_	Stem	Li et al. (2015b)
18	Isopimara-8 (14),15-diene-12α,19-diol	C_20_H_32_O_2_	Stem	Li et al. (2015b)
19	3-Oxo-14,15-dihydroxyabieta-8,11,13-trien-19-ol	C_20_H_28_O_4_	Stem	Li et al. (2015b)
20	Triptobenzene W	C_21_H_30_O_3_	Root	Zheng et al. (2020)
21	Epoxyionone A	C_19_H_28_O_6_	Root	Zheng et al. (2020)
22	Loliolide A	C_17_H_24_O_6_	Root	Zheng et al. (2020)
23	Hypoglicin A	C_20_H_30_O_4_	Stem	Chen et al. (2018b)
24	Hypoglicin B	C_20_H_24_O_3_	Stem	Chen et al. (2018b)
25	Hypoglicin C	C_19_H_20_O_3_	Stem	Chen et al. (2018b)
26	Hypoglicin D	C_20_H_26_O_5_	Stem	Chen et al. (2018b)
27	Hypoglicin E	C_20_H_28_O_2_	Stem	Chen et al. (2018b)
28	Hypoglicin F	C_20_H_28_O_2_	Stem	Chen et al. (2018b)
29	Hypoglicin G	C_20_H_28_O_2_	Stem	Chen et al. (2018b)
30	Hypoglicin H	C_20_H_26_O_3_	Stem	Chen et al. (2018b)
31	Hypoglicin I	C_20_H_26_O_3_	Stem	Chen et al. (2018b)
32	Hypoglicin J	C_20_H_26_O_3_	Stem	Chen et al. (2018b)
33	Hypoglicin K	C_20_H_22_O_6_	Stem	Chen et al. (2018b)
34	Hypoglicin L	C_20_H_22_O_6_	Stem	Chen et al. (2018b)
35	Hypoglicin M	C_19_H_20_O_4_	Stem	Chen et al. (2018b)
36	19-*O*-*β*-D-Glucopyranosyl-labda-8 (17),14-dien-13-ol	C_26_H_44_O_7_	Aerial parts	Zhao et al. (2018)

The majority of diterpenoids exhibit strong immunosuppressive and anti-inflammatory activities. The inhibitory activity towards LPS-induced NO production of these terpenoids was evaluated in microglial BV-2 cells, and all of the compounds showed inhibitory effects (Zhao et al., 2014). Triptolide is a typical representative abietane terpenoid in THH, that has been reported to have diverse pharmacological effects such as anti-inflammatory, antiproliferative, proapoptotic, immunosuppression, and tumour inhibition effects, and this compound exhibits great efficacy in the treatment of rheumatoid arthritis, asthma, and Shin disease (Shamon et al., 1997; Li J. et al., 2012; Chen S.-R. et al., 2018).

### Triterpenoids

Triterpenoids are also one of the main components of THH. The triterpenoids found in THH are mainly pentacyclic triterpenoids, and three types are predominantly noted, including oleanane-type, ursane-type and friedelane-type. Such pentacyclic triterpenoids are active ingredients in the treatment of autoimmune diseases.

One new triterpenoid named 6α-hydroxy triptoline, was isolated from the 95% ethanol extract of THH roots (Song et al., 2021). One friedelane-type triterpenoid (2,3-dihydroxy-6-oxo-D:A-froedo-24 nor-1,3,5 (10), 7-oleanatetraen-29-oic acid) and other types of triterpenoids, including 23-nor-oxopristimerol and hypoglaside A, were isolated from the 95% ethanol extract of THH roots (Li X, 2006). Two oleanane-type triterpenoids, wilforlide A and 3-oxo-oleanoic acid were isolated from the chloroform emulsification layer of THH roots (Wang et al., 2011). One oleanane-type triterpenoid, triptotriterpenic acid A was isolated from 95% ethanol extract of the rhizomes of THH (Ding, 1991). The following triterpenoids were isolated from the methanol extract of THH rhizomes: four oleanane-type triterpenoids, including triptocallic acid D, triptocallic acid C, 3-epikatonic acid, oleanoic acid 3-*O*-acetate; two friedelane-type triterpenoids, 29-hydroxyfriedelan-3-one and polpunonic acid; one ursane-type triterpenoid, 3β-acetoxy-urs-12-ene-28-oic acid; and other types of triterpenoids, such as triptohypol D, triptohypol E, triptohypol F, and hypodiol (Fujita et al., 2000). The following triterpenoids were isolated from the methanol extract of THH roots: one oleanane-type triterpenoid, mesembryanthemoidigenic acid; ten friedelane-type triterpenoids, celastolide, triptoethylol A, wilforic acid A, triptohypol B, triptohypol C, wilforol A, wilforol B, demethylzeylasteral, wilforic acid C, and cangoronine; and other types of triterpenoids, including salaspermic acid and 23-nor-6-oxo-demethyl pristimerol (Duan et al., 1997). Three oleanane-type triterpenoids, including 3-acetoxy oleanolic acid, glut-5-en-3β, 28-diol, and 3-oxo-olean-Δ9(11), 12-diene and two friedelane-type triterpenoids, canophyllal and friedelin were isolated from 95% ethanol extracts of the root bark of THH (Liu et al., 2011) (Table 2). Celastrol, the first triterpenoid ingredient isolated from the genus *Tripterygium*, is classified as a friedelane-type triterpenoid. Its toxicity is much lower than that of triptolide. Celastrol has significant anticancer effects and relieves insulin resistance. Thus, celastrol has attracted considerable attention in recent years (Petronelli et al., 2009; Yang et al., 2014).

**TABLE 2:** Triterpenoids isolated from *Tripterygium hypoglaucum.*

Serial number	Compound	Molecular formula	Plant part	References
37	Celastrol	C_29_H_38_O_5_	Root	Duan et al. (1997)
38	Wilforic acid A	C_29_H_42_O_4_	Root	Duan et al. (1997)
39	Wilforol B	C_29_H_42_O_4_	Root	Duan et al. (1997)
40	Wilforic acid C	C_30_H_48_O_4_	Root	Duan et al. (1997)
41	Celastolide	C_30_H_46_O_5_	Root	Duan et al. (1997)
42	Hypodiol	C_30_H_50_O_2_	Root	Duan et al. (1997)
43	Salaspermic acid	C_32_H_52_O_4_	Root	Duan et al. (1997)
44	Cangoronine	C_30_H_44_O_5_	Root	Duan et al. (1997)
45	Popanonic acid	C_30_H_48_O_3_	Root	Duan et al. (1997)
46	Demthylzeylasteral	C_30_H_38_O_6_	Root	Duan et al. (1997)
47	Mesembryanthemoidigenic acid	C_32_H_52_O_3_	Root	Duan et al. (1997)
48	3-Hydroxy-D: A friedoolean-3-en-2-on-29-oic-acid	C_30_H_46_O_4_	Root	Duan et al. (1997)
49	Triptohypol A	C_30_H_40_O_6_	Root	Duan et al. (1997)
50	Triptohypol B	C_30_H_40_O_5_	Root	Duan et al. (1997)
51	Triptohypol C	C_29_H_39_O_4_	Root	Duan et al. (1997)
52	Triptohypol D	C_32_H_54_O_2_	Root	Fujita et al. (2000)
53	Triptohypol E	C_31_H_52_O_2_	Root	Fujita et al. (2000)
54	Triptohypol F	C_31_H_52_O_2_	Root	Fujita et al. (2000)
55	Regelin	C_31_H_48_O_4_	Root	Zhang et al. (1992a)
56	hypoglaulide	C_30_H_44_O_3_	Root	Zhang et al., (1992a)
57	3-Oxofriedelan-29-oic acid methyl ester	C_32_H_52_O_3_	Root	Yi and Yang (1993)
58	3*β*,22α-Dihydroxy-Δ^12^-ursen-30-oic acid methyl ester	C_30_H_48_O_4_	Root	Yi and Yang (1993)
59	3*β*,22α-Dihydroxy-Δ^12^-oleanen-29-oic acid methyl ester	C_30_H_50_O	Root	Yi and Yang (1993)
60	Friedelin	C_30_H_50_O	Root bark	Liu et al. (2011)
61	3-Oxo-olean-9 (11),12-diene	C_30_H_46_O	Root bark	Liu et al. (2011)
62	Canophyllal	C_30_H_48_O_2_	Root bark	Liu et al. (2011)
63	6α-Hydroxy triptocslline	C_28_H_42_O_5_	Root	Song et al. (2021)

### Alkaloids

Alkaloids are one of the strongest bioactive ingredients found in the THH extract. The alkaloids obtained from THH exhibit pyridine alkaloid structures formed by condensation of dihydroagarofuran-type sesquiterpenes with different pyridinic acids. Studies have shown that alkaloids found in THH from traditional Chinese medicine exhibit antitumour activity in animals.

In 1997, seven alkaloids were isolated from the methanol extract of THH roots: euonine, hyponine A, hyponine B, hyponine C, cangorinine E-I, regelidine, and 3-pyridinecarboxylic acid (Duan et al., 1997). Then, six alkaloids were isolated from the methanol extract of THH roots in 1998: hypoglaunine A, hypoglaunine B, hypoglaunine C, hypoglaunine D, euonymine, and wilforine (Duan and Takaishi, 1998). Two new alkaloids, wilfordine and wilformine were isolated from the ethanol extract of THH roots in 1999 (Li et al., 1999). Moreover, hyponines D, hyponines E, hyponines F, neoeunoymine and forestine were isolated from the methanol extract of THH roots (Duan and Takaishi, 1999). In 2000, six alkaloids were isolated from the methanol extract of THH roots: triptonines A, triptonines B, wilfordinines A, wilfordinines B, wilfordinines C and peritassine A (Duan et al., 2000). In 2012, two sesquiterpene pyridine alkaloids, hypoglaunine E and hypoglaunine F were isolated from 95% ethanol extracts of THH roots (Li X, 2006). Then, four alkaloids, 2-*O*-deacetyleuonine, wilfortrine, triptolide (wilforgine) and tripfordine C were isolated from a 95% ethanol extract of the root bark of THH (Xie et al., 2012). In 2014, four new alkaloid compounds were isolated from the methanol extract of the rhizome of THH: 9α-cinnamoyloxy-1β-furoyloxy-4-hydroxy-6α-nicotinoyloxy-β-dihydroagarofuran, 1β,9α-dibenzoyloxy-4-hydroxy-6α-nicotinoyloxy-β-dihydroagarofuran, 1β-benzoyloxy-9α-cinnamoyloxy-4-hydroxy-6α-nicotinoyloxy-β-dihydroagarofuran and 1β-acetoxy-9α-benzoyloxy-4-hydroxy-6α-nicotinoyloxy-β-dihydroagarofuran (Chen X.-L. et al., 2018) (Table 3). The alkaloid fraction in THH is characterized by high activity and low toxicity; thus, these compounds exhibit potential value in new drug development research.

**TABLE 3:** Alkaloids isolated from *Tripterygium hypoglaucum.*

Serial number	Compound	Molecular formula	Plant part	References
64	Hypoglaunine A	C_41_H_47_O_20_N	Root	Duan and Takaishi (1998)
65	Hypoglaunine B	C_41_H_47_O_20_N	Root	Duan and Takaishi (1998)
66	Hypoglaunine C	C_43_H_49_O_19_N	Root	Duan and Takaishi (1998)
67	Hypoglaunine D	C_41_H_47_O_19_N	Root	Duan and Takaishi (1998)
68	Wilforine	C_43_H_49_O_18_N	Root	Duan and Takaishi (1998)
69	Wilforgine	C_41_H_47_O_19_N	Root	Duan and Takaishi (1998)
70	Wilfordine	C_43_H_49_O_19_N	Root	Duan and Takaishi (1998)
71	Wilfortrine	C_41_H_47_O_20_N	Root	Duan and Takaishi (1998)
72	Euonymine	C_38_H_47_O_18_N	Root	Duan and Takaishi (1998)
73	Wilformine	C_41_H_48_O_18_N_2_	Root	Duan and Takaishi (1998)
74	Wilfornine	C_41_H_47_O_19_N	Root	Duan and Takaishi (1998)
75	Hyponine A	C_41_H_47_O_19_N	Root	Duan et al. (1997)
76	Hyponine B	C_41_H_47_O_19_N	Root	Duan et al. (1997)
77	Hyponine C	C_43_H_49_O_18_N	Root	Duan et al. (1997)
78	Cangorinine E-I	C_43_H_49_O_18_N	Root	Duan et al. (1997)
79	Evonine	C_36_H_43_O_17_N	Root	Duan et al. (1997)
80	3-Pyridinecarboxylic acid	C_29_H_34_O_7_N	Root	Duan et al. (1997)
81	Hyponine D	C_47_H_50_O_18_N_2_	Root	Duan and Takaishi, (1999)
82	Hyponine E	C_45_H_48_O_19_N_2_	Root	Duan and Takaishi (1999)
83	Hyponine F	C_41_H_47_O_19_N	Root	Duan and Takaishi (1999)
84	Neoeunoymine	C_36_H_45_O_17_N	Root	Duan and Takaishi (1999)
85	Forrestine	C_43_H_49_O_18_N	Root	Duan and Takaishi (1999)
86	Peritassine A	C_39_H_49_O_18_N	Root	Duan et al. (2000)
87	Regelidine	C_35_H_37_O_8_N	Root	Duan et al. (2000)
88	Triptonine A	C_45_H_55_O_21_N	Root	Duan et al. (2000)
89	Triptonine B	C_45_H_55_O_22_N	Root	Duan et al. (2000)
90	Wilfordinine A	C_36_H_55_O_12_N	Root	Duan et al. (2000)
91	Wilfordinine B	C_38_H_60_O_13_N	Root	Duan et al. (2000)
92	Wilfordinine C	C_43_H_60_O_14_N	Root	Duan et al. (2000)
93	2-*O*-Deacetyleuonine	C_36_H_45_O_17_N	Root bark	Xie et al. (2012)
94	Tripfordine C	C_36_H_45_O_17_N	Root bark	Xie et al. (2012)
95	1-α-Benzoyloxy-6*β*-nicotinoyloxy-9*β*-acetoxy-4*β*-hydroxy-dihydro-*β*-agarofuran	C_30_H_35_O_8_N	Root	Duan et al. (2000)

### Flavonoids

In 1998, two flavonoids were isolated from industrial alcohol extracts of THH stems: (+) -catechin and *L*-epicatechin (Zhang and Zhang, 1998). In 2012, two flavonoids were isolated from a 95% ethanol extract of the root bark of THH: 4′-O-(−)methyl-epigallocatechin and (2R, 3R)-3,5,7,3′,5′-pentahydroxyflavan (Xie et al., 2012).

### Lignans

Three new lignans, 9′-O-benzoyl-lariciresinol, 9′-O-benzoyl-5′-methoxylariciresinol, and 9′-O-cinnamoyl-lariciresinol, were isolated from the 95% EtOH extract of the twigs of THH. Moreover, only 9′-O-benzoyl-lariciresinol showed weak cytotoxicity against HepG2/Adr cells, with an IC50 value of 30.1 µM *in vitro* (Liu et al., 2011). Recently, syringaresinol, a common bisepoxylignan, was isolated from the 95% EtOH extract of THH (Song et al., 2021).

### Other Compounds

In addition to diterpenes, triterpenes and alkaloids, other compounds, such as steroids, fatty acids, tannins and glycosides, have been identified in THH. Although terpenoids are the main pharmacodynamic components of THH, other components also play synergistic roles.

In 1991, two steroidal compounds, β-sitosterol and daucosterol, and one other compound, fumaric acid, were isolated from the 95% ethanol extract of the rhizome of THH (Ding L, 1991). Two steroidal compounds, ergosta-4,6,8 (14), 22-tetraen-3-one and stigmast-4-en-3-one and three other compounds, *p*-hydroxyl benzoic acid, 3,4-dihydroxy-benzoic acid, and 3-meth-oxy-4-hydroxy-benzoic acid were isolated from the ether extract of THH stems (Zhang and Zhang, 1998). In 2011, three fatty acid compounds, palmitic acid, stearic acid and tricosanoic acid were isolated from the 95% ethanol extract of the root bark of THH (Liu et al., 2011). Two tannins were isolated from industrial alcohol extracts of THH stems: procyanidin B-3 and procyanidin B-4 (Zhang and Zhang, 1998). In 2011, a tannin compound, procyanidin B-2, was isolated from the chloroform emulsified layer of THH roots (Petronelli et al., 2009). Two glycosides, 3,4-dimethoxyphenyl-β-D-glucopyranoside and 3,4,5-trimethoxyphenyl-β-D-glucopyranoside were isolated from the 95% ethanol extract of the root bark of THH (Xie et al., 2012).

### Pharmacological Activity

**FIGURE 4:**
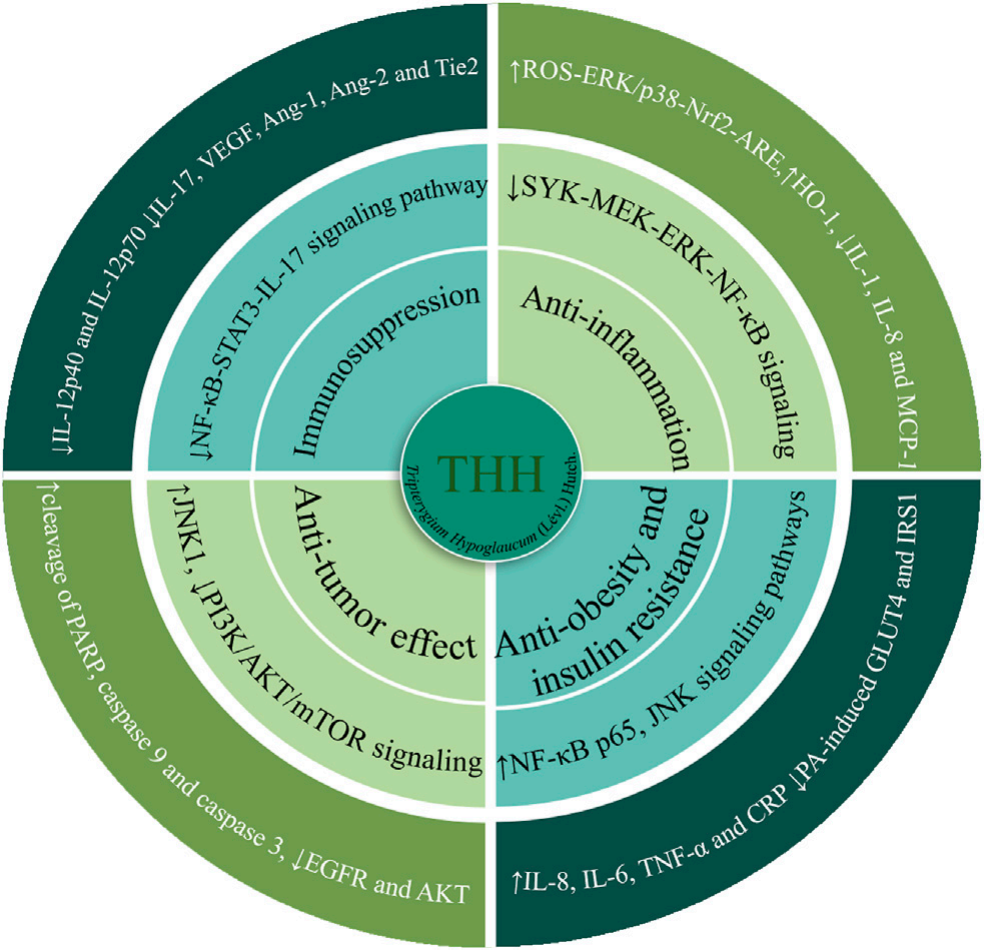
The main signalling pathways and molecular targets of the pharmacologically active components in THH.

THH is a traditional medicine that exhibits various pharmacological activities, including anti-inflammation, immunosuppression, antitumour, obesity and insulin resistance, antifertility, and antiviral effects. These pharmacological effects are mainly related to the alkaloids and terpenoids of THH. These components are expected to exhibit therapeutic significance in various human immunological diseases, such as rheumatoid arthritis, systemic lupus erythematosus, acute infectious hepatitis, chronic nephritis, leukemia, neurodermatitis, and chronic urticaria (Guo et al., 2018; Tang et al., 2020). Here, we summarized several classical pharmacological activities of THH (Figure 4, Tables 4 and 5).

**TABLE 4:** Pharmacological effects of different compounds isolated from *Tripterygium hypoglaucum in vitro* studies.

Pharmacological activity	Compound	Experimental design	Molecular targets/mode of action	References
Anti-inflammatory	THH alkaloids	10 µg of total RNA isolated from THH-treated (40 µg/ml, 8 h) and untreated HL-60 cells	↑genes related to the NF-κB signalling pathway and cell apoptosis (such as NFKBIB, PRG1 and B2M),↓c-myc binding protein and apoptosis-related cysteine proteases caspase-3 and caspase-8	Zhuang et al. (2004)
—	Triptolide	Corneal fibroblasts absence or presence of IL-1 (10 ng/ml), with or without triptolide (30 nM) or dexamethasone (100 nM)	↓IL-1, IL-8 and MCP-1	Lu et al. (2005)
—	Triptolide	Raw 264.7 cells stimulated with LPS (50 ng/ml) in the presence or absence of triptolide (30 µM) for 24 h	↓DNA binding activity of NF-κB, ↓NO production, ↓phosphorylation of c-Jun NH(2)-terminal kinase (JNK)	Kim et al. (2004)
—	Triptolide	Microglia were pre-treated with PBS, triptolide (10, 30, or 50 µM), with or without Bay11-7,082 for 30 min before LPS treatment (10 ng/ml, 24 h)	↓p38-NF-kappaB-COX-2-PGE(2) and JNK-PGE(2)	Gong et al. (2008)
—	Triptolide	TP-mmc (15 μM) with or without TP (1.2, 12, 15, 30 µM) was added to RAW 264.7 cells (middle panel: bar, 50 μM) for 2 h	↓TAK1-TAB1 complex kinase activity	Lu et al. (2014)
—	Celastrol	fibroblast-like synoviocytes (FLSs) were treated with celastrol (0.05, 0.1, 0.2, 0.4 and 0.8 mM) for 24 h	↓MMP-9 promoter activity, ↓TLR4/MyD88/NF-κB pathway, ↓ FLS migration and invasion	Li et al. (2013a)
—	Celastrol	HaCaT cells were incubated with celastrol (0, 0.1, 0.5, 1 µg/ml) for 1h, and stimulated with IFN-c (100 U/ml) for 4 h (for RNA) or 12 h (for protein)	↓IFN-γ-induced ICAM-1 mRNA and protein expression	Seo et al. (2010)
—	Celastrol	HaCaT cells were pretreated with DPI (10 μM), NAC (20 mM) or EUK 134 (50 μM) for 1 h, and then incubated with celastrol (1 μg/ml) for 6 h (for RNA) or 12 h (for protein)	↑ROS-ERK/p38-Nrf2-ARE, ↑HO-1	Seo et al. (2011)
—	Celastrol	RAW264.7 cells were treated with 0–1 μM of celastrol for 24 h	↓ nitric oxide synthase and cyclooxygenase-2, ↓ MPO activity, ↓IL-6 and TNF-α	Kim et al. (2009)
—	Celastrol	BV-2 cells were pre-treated with various concentrations of celastrol (1, 10 and 100 nM) for 30 min prior to stimulation with LPS (10 ng/ml) for 6 h (for TNF-α) and 24 h (for IL-1β)	↓expression of mRNAs of iNOS and cytokines; ↓NO, IL-1β and TNF-α; ↓ERK1/2 phosphorylation and NF-κB activation	Jung et al. (2007)
—	Celastrol	Jurkat T cells were preincubated for 30 min with celastrol (0, 0.3, 1 µg/ml) and followed by the stimulation with of TNF-a (20 ng/ml) or PMA (50 ng/ml) for 90 min	↓IKK activity and IKKβ activity, ↓Bfl-1/A1 expression	Lee et al. (2006)
—	Celastrol	Neutrophils were pre-incubated with different doses of celastrol (0.5–20 μM) or vehicle only at RT for 45 min	↓neutrophil oxidative burst and NET formation, ↓SYK-MEK-ERK-NF-κB signalling cascade	Yu et al. (2015)
Immuno-suppression	Triptolide	Monocytes were cultured for 5 days in the presence of various concentrations (1–20 ng/ml) of triptolide and Dex (10−8–10–6 M)	↓CD1a, CD40, CD80, CD86 and HLA-DR expression; ↑CD14 expression	Zhu et al. (2005)
—	Triptolide	LPS (100 ng/ml)-stimulated U937 cells were treated with or without triptolide (12.5 nM)	↓TREM-1 and DNAX-associated protein (DAP)12, ↓activation of JAK2 and STAT3, ↓TNF-α, IL-1β and IL-6	Fan et al. (2016)
—	Celastrol	Bone marrow-derived macrophages were pre-treated with celastrol (0.1, 0.5 and 1 μM) for 1 h and treated with Alexa Fluor 594 conjugated with LPS (1.5 μg/sample) for 30 min	↓TNF-*α*, IL-6, IL-12, and IL-1*β*, ↓LPS binding to the TLR4/MD2 complex	Lee et al. (2015)
—	Celastrol	Purified CD4+CD25− T cells were treated in the presence or absence (control) of Celastrol (200 nM)	↓mTOR, HIF-1*α*, c-Myc and Akt expression in Th17 cells, ↑FAO of lipids by upregulating CPT1A and AMPKα expression in iTreg cells	Zhang et al. (2018)
—	Triptolide	Confluent synovial cells were treated with recombinant human interleukin-1α (IL-1α; 1 ng/ml), dexamethasone (Dex), and/or triptolide (2.8–140 nM) at the indicated concentrations	↓IL-1α-induced production of proMMP- 1 and -3, ↑IL-1alpha-induced gene expression and production of TIMP-1 and -2	Lin et al. (2001)
—	Triptolide	Human monocytes were cultured for 7 days with G4 medium in the presence of TPT (D2-7, 0.5–10 nM) or medium alone	↓production of IL-12 p70	Chen et al. (2005)
—	Triptolide	C57BL/6 mouse bone marrow cells were cultured with mGM-CSF and mIL-4, and triptolide (0, 1, 5, 10, 20, 50, and 100 ng/ml) added on day 3 of culture (Trip-DC)	↑activation of p38, ↓activation of caspase 3	Liu et al. (2004)
	Triptolide	THP-1 cells were differentiated into macrophage-like cells upon exposure to Me_2_SO, and then cultured with IFN-gamma (500 kU/L) and lipopolysaccharide (LPS) (1 mg/L) with or without triptolide (2.5–0.625 µg/L)	↓CD80 and CD86 expression on IFN-gamma- (500 kU/L) and LPS- (1 mg/L) activated THP-1 cells, ↓IL-12p40 and IL-12p70	Liu et al. (2008)
Antitumour effect	Total alkaloids	JB6 Cl41 cells were suspended containing 10% FBS, 10 μM TPA with or without THHta (1.25–10 μg/ml)	↑activation of caspase-3 and PARP, ↓Bcl-2, Bcl-xL and XIAP	Jiang et al. (2014)
—	Triptonide	treatment of the pancreatic cancer cell lines Patu8988 and Panc1 with triptonide at the doses of 0–20 nM	↓VE-cadherin and CXCL2 genes	Han et al. (2018)
—	Triptolide	PC-3 and DU145 cells were incubated with triptolide (0, 25, 50 nM) for 24 h	↓RNA polymerase activity, ↓CDC25A, and MYC and Src oncogenes	Yuan et al. (2020)
—	Triptolide	Malignant (BxPc-3, MIA-PaCa2 and AsPC-1) and nonmalignant (pancreatic ductal cells and MSC) cells grown under normoxia or hypoxia in the presence or absence of triptolide (20 nM)	↓epithelial-mesenchymal transition (EMT) and CSC features	Liu et al. (2014a)
—	Triptolide	Urothelial cancer cells were treated with CDDP at the concentration corresponding to IC25 (30 mM) with or without 1 h pretreatment of 30 nM triptolide for 12 h	↓CDDP-induced p53 transcriptional activity	Matsui et al. (2008)
—	Triptolide	The JNK1 knockdown cells were treated with triptolide (14–56 nM) for 15 h	↓phosphatidylinositol 3-kinase (PI3K) activity, ↑c-Jun NH(2)-terminal kinase 1 (JNK1)	Miyata et al. (2005)
—	Celastrol	SO-Rb 50 cells were treated with celastrol nanoparticles (0–54.4 µg/ml) and the same dosage of PEG-b-PCL micelles without celastrol for 48 h	↓elevated levels of ALT, AST, ALP and AFP; ↓anti-apoptotic Bcl-2 and Bcl-xl; induced the expression of pro-apoptotic Bax, cytochrome C, PARP and caspases	Li et al. (2012b)
—	Celastrol	A549 cells were treated with celastrol (0–8 µM) at the indicated concentration for 24, 48, and 72 h	↑expression of pro-apoptotic Bax, ↓anti-apoptotic Bcl-2 and Akt phosphorylation	Mou et al. (2011)
—	Celastrol	H1650 cells were collected for apoptosis analysis at 24 h after the treatment of celastrol (0.5, 1, 2, 4 μM)	↓EGFR and AKT	Fan et al. (2014)
—	Celastrol	H1299 cells were treated with or without 4 μM celastrol in the absence or presence of 50 μM Z-VAD for 24 h	↑cleavage of PARP, caspase 9 and caspase 3	Chen et al. (2011)
—	Celastrol	Human myeloma cell line U266 cells were treated with celastrol (0.25 and 0.5 µM) for the indicated times (0–48 h)	↑caspase-3 and NF-κB pathways	Tozawa et al. (2011)
Anti-obesity and insulin resistance	Celastrol	Fully differentiated 3T3-L1 adipocytes were incubated with different doses of oligomycin (5, 10, 20 and 30 µg/ml) and celastrol (5, 10, 20 and 30 μM) in DMSO for 48 h	↓oxidative DNA damage, protein carbonylation and lipid peroxidation	Bakar et al. (2014)
—	Celastrol	C3A human hepatocytes were exposed to various concentration (10–50 nM) of celastrol in the serum-free media for 48 h	↓PA-induced GLUT4 and IRS1	Abu Bakar et al. (2017)
—	Celastrol	Myoblasts were treated with different concentrations (10, 20, 30, 40, 50 and 60 nM) of celastrol were prepared and mixed with 0.1% (v/v) DMSO. The vehicle consisted of an equal amount of DMSO was used as a control	↑NF-κB p65, c-Jun NH(2)-terminal kinase (JNK) signalling pathways,↑IL-8, IL-6, TNF-α and CRP	Abu Bakar and Tan (2017)
Antiviral effect	Triptolide	HONE1/Akata, HK1/Akata, C666–1, and CNE1 cells were placed in 35 mm culture dishes (500 cells/dish) and cultured in standard medium with DMSO control (0.01%) or triptolide (1, 2, or 5 nM) for 2 weeks	↓ratio of Bax/Bcl-2, ↑activated caspase-3 and Nrf2	Zhou et al. (2018)
—	Triptolide	Human umbilical vascular endothelial cells (HUVEC) were trested with or without brusatol (40 nmol/L)/celastrol (50 nmol/L)/AngII(400 nmol/L) for 24 h	↑caspase-9-dependent apoptosis	Li et al. (2017a)
—	Celastrol	The transfected ava5 cells were treated with celastrol (0, 0.2,0.3,0.4,0.5 μM) for 3 days	↓iNOS, TNF-α, and NF-κB phospho-p65 expression, ↑nuclear levels of Nrf2 and HSF-1	Tseng et al. (2017)
Other effects	Triptolide	The model microglial group was treated with Aβ1–40 (20 μg/ml). The low-dose triptolide microglial group was treated with Aβ1–40 (20 μg/ml) and triptolide (5 μg/ml). The high-dose triptolide microglial group was treated with Aβ1–40 (20 μg/ml) and triptolide (25 μg/ml)	↑IL-10, ↓IL-4	Nie et al. (2012)
—	Triptolide	RASF, HM, HFF, or U937 cells were incubated overnight with or without LPS (2 μg/ml) in the presence or absence of the indicated concentrations of T2 (1,2,4 μg/ml), triptolide (1,2,4 ng/ml), DEX (0.1,1,10 μM), or Indo (0.01,0.1 μg/ml)	↓protein levels of inducible nitric oxide synthase (iNOS) and cyclooxygenase-2 (COX-2)	Tao et al. (1998)

**TABLE 5:** Pharmacological effects of different compounds isolated from *Tripterygium hypoglaucum in vivo* studies.

Pharmacological activity	Compound	Experimental design	Molecular targets/mode of action	Duration of treatment	Route of administration	References
Anti-inflammation	Triptolide	Triptolide (8, 16 and 32 mg/(kg day); n = 16, respectively), dexamethasone (1 mg/(kg every 2 days); *n* = 16) or vehicle (*n* = 20)	↓metalloproteinases-13 and -3,↓COX-2 and PGE(2),↓IL-1β, TNF-α and IL-6,↑metalloproteinases-1 and -2	Daily for a period of 21 days	Oral administration	Lin et al. (2007)
—	Triptolide	Triptolide (0.1 mg/kg/d) dissolved in 5% dimethyl sulfoxide was intraperitoneally injected into the SCI rats	↑miR-96, ↓Iba-1 and IKKβ/NF-κB-related proteins, ↓IL-1β and TNF-α	Continued for successive 10 days	Intraperitoneal injection	Huang et al. (2019)
—	Tripterine	tripterine (5, 10 and 20 mg kg−1 day−1), or prednisone (10 mg kg−1 day−1); 0.5% CMC solution as vehicle-treated group	↓ IgG and delayed-type hypersensitivity (DTH), ↓IL-1β and TNF-α	5 days	Intragastrical administration	Li et al. (2008)
—	Celastrol	1 μg/g Celastrol or 2 μg/g digoxin were administrated at adjuvant-induced arthritis (AIA) rats	↓IL-1β and TNF	After 4 days (early treatment group) and after 11 days (late treatment group) of disease induction	Intraperitoneal injection	Cascão et al. (2012)
—	Celastrol	Saline (20 μL) containing 4 μg of sPLA2IIA with celastrol (1, 10, 30 μM and 100 µM)/vehicle was injected into the intra-plantar surface of the right hind footpad of mice	↓sPLA2IIA, 5-LOX and COX-2 enzymes	After 45 min, mice were anaesthetized with pentobarbitone (30 mg/kg, i.p.) and euthanized	Injected into the intra-plantar surface of the right hind footpad of mice	Joshi et al. (2016)
—	Celastrol	Hepa1-6 single-cell suspension cells (2 × 107/ml) were injected subcutaneously at a volume of 0.1 ml in the right flank of each mouse	↓AKT pathway and VEGF autocrine system	21 days of administration	Intraperitoneal injection	Zhang et al. (2019a)
Immuno-suppression	THH	C57BL/6 mice were used to model CIA mice received THH 420 mg/kg/day or the same amount of normal saline (NS)	↓TNF-α, IFN-γ, and IL-17A mRNA and protein levels; ↓NF-κB-STAT3-IL-17 pathway	20 days	Intragastrical administration	Zhou et al. (2020)
—	Celastrol	CIA mice were treated intraperitoneally (IP) with celastrol in phosphate buffered saline (PBS; 3 mg/kg) or PBS alone	↓osteoclastic genes (Trap, Ctsk, Ctr, Mmp-9) and transcription factors (c-Fos, c-Jun and NFATc1), ↓NF-κB and MAPK phosphorylation	15 days	Intraperitoneal injection	Gan et al. (2015)
—	Triptolide	CIA rats were treated with triptolide (11–45 µg/kg/day) starting on the day 1 after first immunization	↓Matrigel-induced cell adhesion of HFLS-RA and HUVEC, ↓TNF-α, IL-17, VEGF, VEGFR, Ang-1, Ang-2 and Tie2, ↓IL1-β-induced ERK phosphorylation and p38 and JNK protein levels	Daily for a period of 28 days from day 1 to day 28 of first immunization	Oral administration intragastrically using syringe feeding	Kong et al. (2013)
—	Celastrol	Female Sprague Dawley rats were treated by celastrol (1 mg/kg/day, i.p.)	↑IL-10, ↓TNF-α, ↓immunohistochemical expression of TLR2 and CD3+ T-lymphocytic count	32 days	Intraperitoneal injection	Abdin and Hasby, (2014)
Antitumour effect	Celastrol	C57BL/6N mice were treated with 1 mg/kg of celastrol, 3 mg/kg of celastrol, or a vehicle control. Celastrol was dissolved in vehicle (10% DMSO, 70% Cremophor/ethanol (3:1), and 20% PBS)	↑ROS-mediated caspase-dependent apoptosis; ↓PI3K/AKT/mTOR signalling	20 days	Oral gavage every 2 days	Lee et al. (2012)
Anti-obesity and insulin resistance	Celastrol	C57BL/6 mice were allowed to recover for 2 weeks postsurgery before receiving intraperitoneal vehicle or celastrol (100 μg/kg) at 6 pm each day	↓TC, TG, LDL-c and Apo B in plasma,↓NADPH oxidase activity	10 consecutive days	Intraperitoneal injection	Kyriakou et al. (2018)
—	Celastrol	Sprague–Dawley rats were treated with celastrol (1.0 ml/100 g) or simvastatin (1.0 ml/100 g)	↑protein phosphorylation of insulin signalling cascades with amplified expression of AMPK protein, ↓attenuated NF-κB and PKC θ activation	6 weeks	Intragastrical administration	Wang et al. (2014)
Antiviral effect	Triptolide	Male BALB/C mice were intravenously (i.v.) treated with a single dose of TP (1.2 mg/kg)	↓TNF-α, IL-1*β*, IL-6 malondialdehyde (MDA) and antioxidative superoxide dismutase (SOD), ↑glutathione (GSH) and glutathione peroxidase (GPx)	24 h	Intravenous injection	Zhou et al. (2014)
Other effects	Celastrol	Celastrol (0.5 and 1.0 mg/kg, i.v.) was administered to anaesthetized rats 2 h before and 30 min after LPS challenge (10 mg/kg, i.v.)	↑Nrf2 activation, ↓Nox2/AT1 receptor expression, ↑phosphorylation of ERK1/2	8 h	Intravenous injection	Wang et al. (2015)

### Anti-Inflammatory Effects

Corticosteroids are widely used as anti-inflammatory drugs, but some serious side effects often occur after long-term use. THH has been shown to be effective in a variety of inflammatory diseases. Under the condition of reasonable control of THH toxicity, THH is expected to play a huge potential as an alternative drug. The therapeutic mechanisms of THH include the inhibition of inflammation and capillary permeability as well as the reduction of infiltrates. Moreover, Hypoglicins B-G and J-M, which are isolated from the stem of THH, inhibited NO production with IC_50_ values ranging from 0.72 to 36.91 μM. Moreover, hypoglicin D and hypoglicin L significantly inhibit the mRNA expression of iNOS at doses of 12.5 and 3.13 μM, respectively (Chen X.-L. et al., 2018). Importantly, the compounds found in THH do not act through the pituitary-adrenal cortex system, so there will be no “rebound” phenomenon after stopping the drug, and even long-term use will not damage the immune system (Zhong et al., 2011), but the toxic accumulation of long-term use must be considered carefully.

The mechanism by which triptolide attenuates the inflammatory pathology of Crohn’s disease (CD) involves increasing IL-10 levels and Foxp3+ Tregs in the mucosa and decreasing TNF-α levels in the mucosa (Li G. et al., 2014). The pathogenesis of CD is mainly mediated by the TLR/NF-κB signalling pathway. Triptolide upregulates TLR2 and TLR4 in a CD model in IL-10-null (IL-10 −/−) mice by inhibiting TLR/NF-κB signalling pathways *in vivo*. TLR activation also promotes angiogenesis mediated by exogenous and endogenous ligands in different inflammatory settings (Harmey et al., 2002; Oda and Kitano, 2006; Yu et al., 2010). A key regulator of the signal transduction cascade activated by NF-κB and activator protein-1 is TAK1. However, for TAK1 to be fully activated, it must be induced by a protein activator, TAK1 binding protein (TAB1). Triptolide interferes with the formation of the TAK1TAB1 complex and thus inhibits the activity of TAK1 kinase. The interaction region between triptolide and TAB1 involves the amino acid residues between TAB1373 and 502. Triptolide inhibits MAPK pathway activation in macrophages upon binding to TAB1 (Lu et al., 2014). In addition, triptolide inhibited the activation of microglia and the secretion of inflammatory factors in BV2 cells by upregulating miR96 (Huang et al., 2019). Moreover, the γ-butyrolactone and C-14 β-hydroxyl portions of the triptolide molecules are important components responsible for the compound’s anti-inflammatory characteristics and cytotoxicity (Wong et al., 2008). Triptolide suppresses the production of IL-6, IL-8, TNF-α, and IL-1β in human bronchial epithelial cells, and inhibits staphylococcal exotoxin-stimulated T-cell proliferation. Triptolide induces the expression of monocyte chemotactic protein (MCP)-1, IFN-g, TNF, IL-6, IL-1β, macrophage inflammatory protein (MIP)-1β and MIP-1α in peripheral blood mononuclear cells (PBMCs) (Krakauer et al., 2005).

Angiogenesis is a fundamental event in inflammation. Infection leads to inflammation and growth factors released by leukocytes promote angiogenesis. Many chronic inflammatory diseases, such as retinopathy, rheumatoid arthritis, Crohn’s disease, atherosclerosis, diabetes and cancer are closely associated with angiogenesis (McDonald, 2001; Rodríguez-Martínez et al., 2006; Costa et al., 2007). Celastrol achieves its effect mainly by regulating various inflammatory mediators (Li et al., 2008; Li G. et al., 2013), inhibiting the production of proinflammatory cytokines (IL-6, IL-2, IFN-γ, TNF, IL-1β, IL-8, etc.,) and proinflammatory enzymes (COX-1, prostaglandin E2, NOS, COX-2 and iNOS, etc.,) through inhibition of NF-κB (Lee et al., 2006; Jung et al., 2007; Kim et al., 2009; Seo et al., 2010, 2011; Cascão et al., 2012; Joshi et al., 2016). In addition, celastrol interferes with the migration, proliferation, and activation of inflammatory cells (Yu et al., 2015). Celastrol suppresses the LPS-induced IκB kinase (IKK)/NF-κB pathway, significantly downregulates TLR4 expression and inhibits VEGF secretion in LP-1 cells. These results suggest that celastrol inhibits LPS-induced angiogenesis by inhibiting TLR4-triggered NF-κB activation (Zhang R. et al., 2019).

### Immunosuppression

THH was used to treat various autoimmune diseases based on its action against metabolic pathways, and three metabolites, urocanate, L-glutamate and alanine played very important roles (Guo et al., 2018). The key molecular mechanism of THH against RA is related to inhibition of the inflammatory response through inactivation of the TNF and NF-κB signalling pathways (Jiang et al., 2020b). THH reduces the expression of related inflammatory cytokines in joint tissue and serum, and the mechanism of THH in the treatment of collagen II-induced arthritis (CIA) mice involves inhibition of the NF-κB-STAT3-IL-17 pathway (Zhou et al., 2020). The formation and function of osteoclasts in CIA mice are directly inhibited by celastrol. Celastrol significantly reduced joint bone damage, and inhibited arthritis, and this activity is associated with the expression of the osteoclast marker serum tartrate-resistant acid phosphatase (TRAP) 5b and osteoclastic genes (Ctr, Mmp-9, Ctsk and Trap) and transcription factors (NFATc1 c-Jun and c-Fos). Moreover, celastrol suppressed bone resorption activity and the formation of TRAP + multinucleate cells in a dose-dependent manner (Gan et al., 2015).

Triptolide and celastrol are the most important active ingredients of THH against RA, and their molecular mechanisms involve many targets, such as inhibition of inflammation, inhibition of angiogenesis, immunosuppression, protection of cartilage, and induction of apoptosis (Kong et al., 2013; Fan et al., 2016, 2018; Wang S. et al., 2018). The expression of chemokines and inflammatory cytokines in RA synovioblasts is inhibited by celastrol through modulation of the OPG/RANKL axis, thereby inhibiting RA inflammation and bone erosion (Feng et al., 2013). Celastrol also decreases the expression of NF-κB, nitrite levels, and TLR2 expression and CD3^+^ Tlymphocyte counts as assessed by immunohistochemistry; and the cytokine phenotype changes significantly from Th1 to Th2, with increased IL-10 and decreased TNF-α levels. (Abdin and Hasby, 2014). In addition, the binding of LPS to the TLR4/MD2 complex and TLR4 activation were also inhibited by celastrol in a thiol-dependent manner (Lee et al., 2015). Celastrol suppresses the expression of phosph-stat3, c-Myc, Glut1, mTOR, HK2, HIF-1a and Akt in Th17 cells; upregulates the expression of phosph-stat5 in iTreg cells, and upregulates the expression of CPT1A and AMPKα in iTreg cells to promote fatty acid-oxidation (Zhang et al., 2018).

Triptolide inhibits the production of proMMP-1 and -3 induced by IL-1α in human synovial fibroblasts and reduces the messenger RNA levels of IL-1α. In contrast, the IL-1alpha-induced gene expression and production of TIMP-1 and-2 were further augmented by triptolide in synovial cells. Triptolide also inhibited the IL-1alpha-induced production of PGE2 by selectively suppressing the gene expression and production of COX-2, but not COX-1 (Lin et al., 2001) (Hu et al., 2004). Triptolide inhibits the production of IL-2, IL-4, and IFN-γ and the activation of CD4^+^ and CD8^+^ T cells in peripheral blood in RA patients, thus exerting an immunosuppressive effect (Ren et al., 2010) (Nong et al., 2019). Professional antigen presenting cells (APCs), such as dendritic cells (DCs), are another target for the immunosuppressive activity of triptolide (Chen et al., 2005). DCs are induced by high concentrations (20 ng/ml) of triptolide via caspase3 activation and sequential p38 MAP kinase phosphorylation (Liu et al., 2004; Liu et al., 2006). Triptolide also inhibits T cells and DC-mediated chemoattraction of neutrophils by reducing NF-κB activation and STAT3 phosphorylation (Zhu et al., 2005). Triptolide prevents the differentiation of immature monocyte-derived DCs (MoDCs), mainly by reducing the ability of MoDCs to stimulate lymphocyte proliferation in allogeneic mixed lymphocyte reactions (MLRs) and downregulating CD86, CD40, CD1a, CD80, and HLA-DR expression. Triptolide reduces IFN-γ-induced expression of CD80 and CD86 in DCs and THP-1 cells (Liu et al., 2005). Recently, it was found that triptolide inhibited IL-12/IL-23 expression in APCs *via* CCAAT/enhancer-binding protein α (Liu et al., 2008).

### Antitumour Effects

The alkaloid fraction of THH induced apoptosis at lower concentrations and significantly inhibited tumour growth *in vitro* and *in vivo*. In addition, the apoptotic effect of total alkaloids in tumour cells is potentially mediated through both the extrinsic death receptor pathway and the intrinsic mitochondrial pathway. The total alkaloids fraction of THH induced apoptosis by inhibiting Bcl-xL, Bcl-2, and XIAP and activating PARP and caspase-3, thereby inhibiting the growth of colon cancer cells in a dose-dependent manner *in vitro* (Jiang et al., 2014). Triptonide potently inhibited vasculogenic mimicry mediated by pancreatic cancer cells (Han et al., 2018). In addition, triptonide inhibited the epithelial-mesenchymal transition of gastric cancer-associated fibroblasts (GCAFs) by correcting abnormalities in microRNA expression and subsequently eliminating the epithelial-mesenchymal transition in gastric cancer cells (Wang et al., 2017). Moreover, a cytotoxicity of triptolide and 2-epilactone was observed, particularly in A549, DU145, KB, KBvin, and MDA-MB-231 human tumour cell lines, with IC_50_ values of 0.0012–0.1306 μM *in vitro* (Li X.-L. et al., 2015). The mRNA and protein levels of CD147 and MMPs were reduced, and triptonide also inhibited the expression of caveolin-1, thereby inhibiting the invasion and migration of prostate cancer cells (Yuan et al., 2020).

Pancreatic cancer (PC) cell lines SNU-213, Capan-1 and Capan-2 exhibit different sensitivities to triptolide with IC_50_ values of 0.01, 0.02, and 0.0096 μM, respectively. Phosphorylation of Chk2 was more significant in SNU-213 sensitive to triptolide but, not in SNU-410 insensitive to triptolide, suggesting that the effect of triptolide on different PC cell lines may be related to gene expression (Liu L. et al., 2014; Kim et al., 2018). In addition, triptolide inhibited CRC growth through induction of cleavage and perinuclear translocation of 14-3-3e protein (Liu et al., 2012). Furthermore, triptolide dose-dependently inhibited the proliferation of human scale-cell carcinoma SAS, human cervical carcinoma SKG-II, and human fibrosarcoma HT-1080 cells, an important target molecule in the antitumour effect of triptolide is phosphatidylinositol 3-kinase (PI3K). The reduction of PI3K activity leads to inhibition of tumour cell proliferation, which subsequently leads to enhanced JNK1 phosphorylation through Akt and/or PKC-independent pathways (Miyata et al., 2005).

As an important compound of THH, celastrol exhibits tumour-inhibiting effects in a variety of tumour models, and its antitumour mechanisms have been demonstrated in various cancers *in vitro* (Li Z. et al., 2012; Lee et al., 2012). There are two main pathways by which celastrol induces apoptosis in cancer cell lines, namely, through the activation of intrinsic pathways (mitochondrial pathways) and extrinsic apoptotic pathways (death receptor pathways) (Mou et al., 2011). Apoptosis, paraptosis, and necrosis are modes of cell death induced by celastrol (Chen et al., 2011); the expression of ERα target genes was suppressed, celastrol also decreased the expression of ERα at the protein and mRNA levels in MCF7 and T47D human breast cancer cells, leading to cell cycle arrest and inhibition of breast cancer cell growth (Jang et al., 2011). Moreover, celastrol induced apoptosis by cleaving the PARP proteins, caspase-3, caspase-9, and caspase-8; increasing FasL and Fas expression; decreasing mitochondrial membrane potential; upregulating proapoptotic Bax expression; and downregulating antiapoptotic Bcl-2 (Mou et al., 2011). In addition, the activation of caspase-3 also involved in the induces myeloma cell apoptosis, and celastrol inhibited NF-κB migration into the nucleus, and treatment of the human myeloma cell line U266 with celastrol induces cell cycle at the G1 phase followed by apoptosis (Tozawa et al., 2011). Moreover, celastrol suppressed the migration of AGS and YCC-2 cells in gastric tumour induced G1 arrest in cell-cycle populations, and increased phosphorylated AMPK levels after reducing the levels of AKT, mTOR, and S6K phosphorylation (Lee et al., 2014). In addition, celastrol can significantly reduce tumour nodules in the liver by decreasing DEN-induced MDM2 levels and activating the p53 pathway, thereby improving DEN-induced liver pathology (Chang et al., 2016). It also induces apoptosis in Bel-7402 cells by the mitochondrial apoptosis pathway (Li P.-P. et al., 2015). Moreover, it upregulates both the mRNA and protein levels of DR4 and DR5, which induce TRAIL/Apo-2 L-mediated apoptosis in cancer cells (Zhu et al., 2010; Lin et al., 2017).

In the last 10 years, given the increased accessibility of oncometabolites, the idea that cancer is a metabolic disorder has gradually emerged (Collins et al., 2017). In reports on colon cancer, many oncometabolites such as histidine, isoleucine, phenylalanine, tyrosine, threonine, glutamate, and pyruvate have been found to be closely related to cancer-associated metabolic pathways (Mishra and Ambs, 2015; Wishart et al., 2016). Treatment with triptolide may help to restore normal metabolite levels in these pathways. Triptolide increases isoleucine, plasma proline, methionine, 2-hydroxyisovalerate (2-HIV), low-density lipoprotein/very low-density lipoprotein (LDL/VLDL) and 2-hydroxyisobutyrate (2-HIB) levels, and reduces plasma pyruvate, 3-hydroxybutyric acid, and glutamine levels. Because 2-HIV, LDL/VLDL and 2-HIB are related to branched-chain amino acid metabolism, lactate and pyruvate metabolism, and cholesterol metabolism, respectively, the antitumour mechanism of triptolide may rely on correcting alterations in serine/glycine/methionine biosynthesis, branched-chain amino acid metabolism, and ketone body metabolism (Li et al., 2019).

### Anti-Obesity and Insulin Resistance Effects

Celastrol targets mitochondria, inhibits the inflammatory response and prevents obesity induced by specific diets in mice. Celastrol-administered mice at 100 μg/kg decrease food consumption and body weight *via* a leptin-dependent mechanism (Kyriakou et al., 2018). In 2015, the Umut Ozcan team at Harvard Medical School screened more than 1,000 compounds that reduce ER stress (insulin resistance is a major risk factor for obesity, and ER stress is an important cause of resistance) and found that celastrol can be used as a weight-loss drug. Obese mice treated with celastrol lost 45% of their body weight, and improved insulin sensitivity was observed. Thus, celastrol may exhibit potential therapeutic effects in various diseases, such as type 2 diabetes and fatty liver (Liu et al., 2015). The weight-loss mechanism of celastrol is mediated through interleukin 1 receptor 1 (IL1R1) (Feng X. et al., 2019), it has no effects on weight loss, diabetes or fatty liver in mice if IL1R1 is absent.

Mitochondrial dysfunction and inflammation tend to occur at high levels of fatty acid saturation in insulin-sensitive tissues (Hernández-Aguilera et al., 2013). Celastrol reduces the body weight of obese mice induced by a high leptin diet by up to 45%, increases insulin sensitivity and energy expenditure, and inhibits food intake. Celastrol is a leptin sensitizer, but it has shown effects in leptin-deficient (ob/ob) and leptin receptor-deficient (db/db) mouse models (Liu et al., 2015). Palmitate-mediated insulin resistance could be blocked by celastrol by improving metabolic activity associated with mitochondrial function (Bakar et al., 2014). In addition, celastrol significantly reduced mitochondrial superoxide production; enhanced the cellular fatty acid oxidation rate, intracellular ATP content and mitochondrial membrane potential citrate synthase activity; increased levels of tricarboxylic acid cycle intermediates; improved mitochondrial function; increased mitochondrial DNA content, and inhibited oxidative DNA damage, and glucose uptake activity and enhances the expression of AMPK and GLUT4 proteins were also improved by decreasing the activation of NF-κB and PKCθ (Abu Bakar et al., 2015; Abu Bakar and Tan, 2017).

Importantly, Celastrol showed an anti-IR activity, it was achieved by reducing miR-223, and celastrol re-upregulated the downregulation of miR-150 and miR-223 in HepG2 cells (human hepatocellular carcinoma cell lines) through the GLUT4 pathway, reversing palmitate-induced insulin resistance (Zhang X. et al., 2019). Celastrol treatment significantly enhanced tyrosine-612 phosphorylation of IRS-1 protein and decreased serine-307 in C3A hepatocytes, protecting cells from mitochondrial dysfunction and insulin resistance (Abu Bakar et al., 2017). Meanwhile, it also reduces serum malondialdehyde (MDA) and reactive oxygen species levels, increases antioxidant enzyme activities, reduces oxidative stress and improves lipid metabolism in a dose-dependent manner by alleviating oxidative damage, and inhibiting NADPH oxidase activity by improving ABCA1 expression. These actions inhibit increases in body weight and alleviate cardiovascular damage (Abu Bakar and Tan, 2017). The inhibition of negative regulators protein tyrosine phosphatase (PTP) 1B (PTP1B) and T cell PTP (TCPTP) in the hypothalamic arcuate nucleus (ARC) were also invovled in body weight reduction. The reversible non-competitive binding of celastrol to these proteins is mediated by an allosteric pocket close to the active site (Kyriakou et al., 2018).

### Antifertility Effect

In 1986, reversible anti-fertility effects of TwHF extract in male rats were discovered, sparking global interest. The researchers performed bioassay-guided subfractionation of material extracted from plants and isolated a series of six male antifertility diterpene epoxides: triptolide, tripdiolide, triptolidenol, tripchlorolide, 16 hydroxytriptolide and T7/19 (Zhen et al., 1995). At the ED95 dosage levels, metamorphic spermatids as well as testicular and epididymal sperm excoriation and late spermatid basic nuclear protein turnover were inhibited. In addition, treatment with these compounds delayed spermatogenesis and sperm head-to-tail separation as well as microtubule, microfilament, and membrane damage (Zhen et al., 1995). Triptolide inhibited GATA4-mediated glycolysis by suppressing Sp1-dependent PFKP expression in SCs and induced testicular toxicity (Zhang et al., 2021b).

Oral THH reversibly induces infertility in men without significant side effects and does not affect libido or potency (Qian et al., 1988). Triptolide induced complete infertility in adult rats with minimal adverse effect on the testis, and it mainly targets epididymal sperm. No effects on testicular and accessory organ weights, tubular lumen volume and total number of Leydig cells, spermatogonia, tubule diameter, and number of Sertoli cells, pachytene (P) spermatocytes, and preleptotene (PL) were observed. Triptolide significantly reduced tubule volume and the number of round spermatids, making it an attractive lead agent as a post-testicular male contraceptive (Lue et al., 1998). We look forward to more reliable data to verify this possibility.

### Antiviral Effects

Many natural products have been found to effectively inhibit unique enzymes and proteins that are essential for the viral life cycle (Vlietinck and Vanden Berghe, 1991; Baker et al., 1995). Hypoglaunine A, hypoglaunine B, triptonine A, and hyponine E have represented compounds extracted and purified from THH roots with anti-HIV viral effects (Cheng et al., 2002). The total alkaloid extracts of THH showed potent anti-HSV-1 activity and low cytotoxicity in Vero cells (Tao et al., 2007). Triptobenzene J and quinone 21 have anti-A/PR/8/34 (H1N1) influenza virus (oseltamivir-resistance) activity with EC_50_ values of 38.6 ± 10.7 μM, and 22.9 ± 6.4 μM, respectively. Quinone 21 exhibited anti-A/Hong Kong/8/68 (H3N2) influenza virus (sensitivity) activity with an EC_50_ value of 21.6 ± 0.6 Μm (Wang et al., 2020).

Moreover, triptolide treatment inhibited macrophage infiltration; inhibited the overproduction of malondialdehyde (MDA), IL-6, TNF-α and IL-1β in reperfusion myocardial tissue; and upregulated the activities of glutathione peroxidase (GPx), antioxidant superoxide dismutase (SOD), and glutathione (GSH), and the nuclear accumulation of Nrf2 and the activity of its downstream target haem oxygenase-1 (HO-1) were enhanced in ischaemic myocardial tissues (Yu et al., 2016). Additionally, triptolide significantly inhibited Epstein-Barr virus (EBV)-positive cell-induced tumour proliferation *in vivo*, and low-dose triptolide significantly decreased the half-life of EBV nuclear antigen 1, obviously reduced the expression of EBV nuclear antigen 1, and enhanced triptolide-induced apoptosis by activating the proteasome-ubiquitin pathway (Zhou et al., 2018). However, mitochondrial dysfunction, apoptotic damage, oxidative stress, and histopathological changes in the heart were noted in normal BALB/C mice treated with 1.2 mg/kg triptolide (Zhou et al., 2014). Therefore, the determination of safe dose is the key to the clinical application of triptolide.

Celastrol also showed effective activities in antivirals, it upregulated ERK1/2 phosphorylation, inhibited reactive oxygen species (ROS) generation, and improved endothelial cell activity and Ang II-mediated HUVEC injury by activating Nrf2 (Li M. et al., 2017); and the nuclear levels of Nrf2, nuclear levels of HSF-1 and cardiac GSH levels are also increased (Wang et al., 2015). Moreover, celastrol also inhibited hepatitis C virus (HCV) replication in both the HCV subgenomic and HCVcc infection systems with EC_50_ values of 0.37 ± 0.022 mM and 0.43 ± 0.019 mM, respectively, the NS3/4A protease activity is inhibited, and further enhancing anti-HCV activity, thereby blocking HCV replication. Interestingly, celastrol showed synergistic effects in combination with the NS5B inhibitors sofosbuvir and interferon-α and the NS5A inhibitor daclatasvir targeting the JNK/Nrf2/HO-1 axis with celastrol presents a promising strategy against HCV infection that could serve as a potential supplement to block HCV replication (Tseng et al., 2017). This result suggests that synergistic use could be a topic in future research.

### Other Effects

THH promoted the expression of Foxp3, increased the level of CD4+/CD25 + T cells, decreased the occurrence of aGVHD, and prolonged survival time in mice by regulating cytokine secretion (Li Z. Y. et al., 2013). Syringaresinol has significant protective activity against excitatory damage induced by sodium glutamate in SH-SY5Y neurons, and the mechanism potentially involves antioxidative stress and repair of mitochondrial function and DNA damage to significantly reduce sodium glutamate-induced neuronal apoptosis (Yan et al., 2019).

In recent years, researchers have gradually started to pay attention to the effect of triptolide in AD. Regarding the neuroinflammatory pathology of the AD brain, triptolides potentially exert neuroprotective effects on synapses by partially inhibiting the mechanism of the Aβ-induced immunoinflammatory response, which may effectively slow the neurodegenerative process of AD (Nie et al., 2012). Triptolide inhibits Aβ-induced elevation of IL-1β and TNF-α levels in cultured rat microglia (Jiao et al., 2008). Triptolide promotes synaptophysin expression in hippocampal neurons in an AD cell model (Nie et al., 2012). Tripchlorolide, a novel analogue of triptolide, protected neuronal cells by blocking microglial inflammatory responses to oligomeric Abeta (1–42), inhibited nuclear translocation of nuclear NF-κB without affecting I-κBα phosphorylation and inhibited Aβ-induced JNK phosphorylation, but not ERK or p38 MAPK (Pan et al., 2009). Triptolide also inhibits the upregulated expression of prostaglandin E2 and IL-1β in lymphocytes and AD cell models (Tao et al., 1998; Li et al., 2011).

### Pharmacokinetics

Over the years, scholars have conducted numerous studies on the pharmacodynamics of THH, but studies on the pharmacokinetics of THH compounds in animals, especially those administered intravenously, are limited. Antitumour pharmacodynamics and toxicological studies of triptolide are closely related to the pharmacokinetics and tissue distribution of triptolide, which are important components of new drug evaluation studies. Oral triptolide is rapidly and highly absorbed and distributed in the liver, heart, spleen, lung and kidney. Biotransformation of triptolide in rats includes hydroxylation, sulfate, glucuronide, N-acetylcysteine (NAC) and glutathione (GSH) conjugation and combinations of these pathways. Less than 4% of triptolide was recovered from the faces, bile and urine within 24 h. After repeating the dosage, triptolide was eliminated quickly without accumulation *in vivo*. As a substrate for P-glycoprotein (P-gp) and CYP3A4, triptolide could have clinically significant pharmacokinetic interactions with protein substrates/inhibitors (Song et al., 2019). After injection of three doses (100 µg/kg, 200 µg/kg, 300 µg/kg) of triptolide into the veins of rats, the corresponding t_1/2α_ of each subsequent dose group was: 0.033, 0.021, and 0.026 h, respectively, and the t1/2β values were 0.753, 0.630, and 0.574 h. The AUC was related to the dose, and the compartmental model was a two-compartment model. After intravenous injection of an effective dose (200 µg/kg) of triptolide in rats, rapid and extensive tissue distribution was observed. After 5 min of administration, the highest triptolide levels were noted in lung tissue followed by liver, kidney, heart, brain, spleen, small intestine, gonads, skeletal muscle, and stomach. Fifteen min after administration, the drug concentrations in all tissues decreased with higher concentrations in the lung, kidney, heart and liver. The distribution order is similar to that noted 5 min after administration. One hour after administration, the drug concentration in each tissue decreased significantly, and high drug concentration was still maintained in the liver and small intestine (Shao et al., 2007).

Celastrol exhibited linearity in the concentration range of 0.05–5 µg/ml with R^2^ of 0.999. Rats were intravenously (IV) administered 1 mg/kg of pure CL in PEG 300 solution, which resulted in a maximum concentration (C_max_) value of 0.17 µg/ml at 5 min following administration (Onyeabor et al., 2019). After oral administration of *Tripterygium* wilfordii tablets (1 tablet/kg) to beagle dogs, the changes in plasma celastrol levels followed a one-compartment model (w = 1/cc), and the main pharmacokinetic parameters were C_max_ (35.64 ± 9.540) μg·L^−1^, Tmax (2.62 ± 0.69) h, T1/2 (2.93 ± 0.29) h, CL (0.308 ± 0.056) Lkg −1·h−1, AUC0–12 (131.16 ± 31.94) μg·L·h−1, and AUC0–∞ (142.83 ± 37.57) μg·L·h−1 (Zhang et al., 2016). Glycyrrhizin significantly decreased the plasma concentration (from 64.36 ng/ml to 38.42 ng/ml) and AUC0-t (from 705.39 to 403.43 μg h/L) of celastrol in rats, and increased the efflux ratio of celastrol (4.02 vs. 6.51). Moreover, glycyrrhizin significantly increased the intrinsic clearance rate of celastrol from 20.3 ± 3.37 to 38.8 ± 4.18 μL/min/mg proteins (Yan et al., 2017).

### Toxicity

**FIGURE 5:**
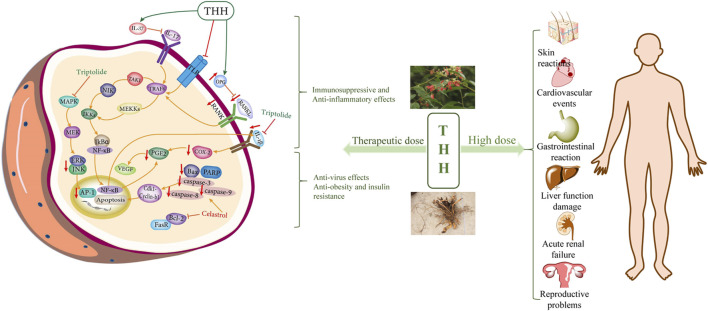
Illustration of the main signalling pathways involved in THH-based therapy and organ toxicities observed at high doses.

The targets of toxicity were closely associated with the p53 signalling pathway, PI3K-Akt signalling pathway and other pathways. Although THH has multiple phytochemical activities and pharmacological effects, it is also limited in clinical application given its toxicities associated with multiple targets (Xu et al., 2004). Although triptolide has attracted remarkable efficacy against diseases, the safety margin of the triptolide medication dose is very narrow, and the accumulation of triptolide in the body can lead to severe hepatotoxicity, renal toxicity, and reproductive toxicity (Sun et al., 2013; Kong et al., 2015; Ma et al., 2015). Therefore, dose control is particularly important in clinical treatment (Figure 5).

Triptolide mainly causes abnormal purine and pyrimidine metabolites in the body through the metabolism of hypoxanthine, allantoic acid, ADA and other proteins. The mechanism of triptolide-induced liver injury exhibits an important relationship with oxidative stress, and excessive ROS and anionic peroxide production, depletion, autophagy, hepatocyte apoptosis, and suppression of antioxidant enzyme activities are the main causes of oxidative stress (Fu et al., 2011; Xi et al., 2017; Wei et al., 2019). Thus, hepatocyte injury is mainly caused by high levels of ROS and reactive nitrogen species (RNS) inhibiting sodium, ATPase, and calcium pump activities in the cell membrane, resulting in decreased mitochondrial membrane potential (MMP) and release of the apoptosis factor caspase-3 (Li J. et al., 2014; Wang Y. et al., 2018; Tabeshpour et al., 2018; Feng Z. et al., 2019; Yuan et al., 2019b; Hasnat et al., 2019). Moreover, triptolide significantly increased serum ALP, AST, and ALT levels; increased hepatic MDA, IL-6, TNF-α, IFN-γ, and IL-1β levels, induced nuclear translocation of NF-κB; and decreased hepatic GSH-Px, CAT, and SOD activities (Yang et al., 2017). It also regulates the metabolism of sphingolipids and glycerophospholipids by affecting metabolites and key proteins such as sphingosine, PC, PKC, PAH, LPC, and PLC, which cause disturbances in Ca^2+^ channels and stimulate oxidative stress levels, leading to kidney damage (Xie et al., 2020). Therefore, reducing liver toxicity and nephrotoxicity through chemical modification is an important topic for triptolide to be applied.

Triptolide induced cytotoxicity in L02 cells, caused mitochondrial dysfunction and altered mitochondrial dynamics, mainly characterized by an imbalance in mitochondrial fusion and fission protein mitochondrial fragmentation. Mitochondrial fragmentation can lead to increasing autophagic flux by triptolide. Changes in mitochondrial dynamics were associated with increasing expression of the Drp1 fission protein. Despite no significant effect on L02 cells, triptolide doses of 12.5, 25 and 50 nM were significantly toxic. Mdivi-1 is a potent mitochondrial fission inhibitor that reverses mitophagy by inhibiting mitochondrial fission and increasing mitophagy *in vitro* and *in vivo* (Hasnat et al., 2019). Treatment of mice with the PPARα agonist fenofibrate alleviated triptolide-induced liver injury, whereas the PPARα antagonist GW6471 increased hepatotoxicity and increased long-chain acylcarnitine through activation of the NOTCH-NRF2 pathway to protect against triptolide-induced liver damage (Hu et al., 2019). The hepatotoxicity of triptolide is closely related to its dose, indicating that the liver is more sensitive to other drugs (CYP450 inhibitors) and their accumulation. Thus, celastrol, which is also a hepatotoxic component of THH and an inhibitor of CYP450, could aggravate triptolide-induced hepatotoxicity, which may explain why the combination of celastrol and triptolide causes more severe liver injury. In summary, celastrol and triptolide damaged primary rat hepatocytes by decreasing cell viability, enhancing cellular stress, decreasing MMP, increasing LDH and ROS levels, and inevitably damaging the cell membrane (Jin C. et al., 2015; Jin et al., 2019).

However, Arctiin exerts antioxidant effects mediated by the Nrf2/ARE pathway and alleviates triptolide-induced hepatotoxicity, and its molecular mechanism may be related to its antioxidative stress ability (Jin J. et al., 2015; Wei et al., 2019). A novel mechanism of triptolide-induced hepatotoxicity is mitochondrial fission associated mitophagy, and triptolide-induced hepatotoxicity can be targeted by mitochondrial fission and mitochondrial autophagy signalling pathways (Hasnat et al., 2019). Specifically, 18β glycyrrhetinic acid (GA) is the main bioactive component of liquorice (*Glycyrrhiza glabra* L.), and low-dose GA (50 mg/kg) effectively reduces triptolide-induced hepatotoxicity in rats through its inflammatory, antioxidative, and antiapoptotic effects (Yang et al., 2017). GA reduced the accumulation of triptolide in HK-2 cells, which were related to P-gp (Li Z. et al., 2017). Pretreatment with GL significantly accelerates the metabolic elimination of triptolide from the body mainly through induction of hepatic CYP3A activity (Tai et al., 2014). Thus, the combination of triptolide with other natural compounds may be an effective measure to control its toxicity and ensure its safety, there still need more further research to investigate its mechanism of combination.

## Discussion and Conclusion

Traditional Chinese medicine (TCM) has unique therapeutic advantages in the treatment of a variety of complex diseases and major diseases, such as cholestasis, nonalcoholic fatty liver disease, liver fibrosis, and ischaemic stroke (Jiang et al., 2020a; Hu et al., 2020). The genus *Tripterygium* contains only 3 species, *i.e. Tripterygium wilfordii* Hook. f., *Tripterygium hypoglaucum* (H. Lév.) Hutch. and *Tripterygium regelii* Sprague and Takeda. In the last 10 years, many studies have discussed the plants related to the genus *Tripterygium*. Among them, *Tripterygium wilfordii* Hook. f., as one of the widely reported plants, has occupied almost all the focus of the review of the genus *Tripterygium*. Individual scholars have included the chemical constituents and pharmacological activities of THH in their studies on the genus *Tripterygium* (Lv et al., 2019)*.* In contrast, our study separately investigated the related active components and pharmacological effects of THH, providing more selectivity for the extraction of components from the genus *Tripterygium*. In addition, we updated the recently published experimental studies and supplemented the pharmacokinetic studies of compounds in THH, so as to provide some ideas for the development of new drugs.

In this review, we summarized the various bioactive components of THH, such as sesquiterpenes, diterpenes, triterpenoids, flavonoids, and lignans. In this review, we introduced the structural grouping of 120 secondary metabolites of THH and described the activity data obtained from THH research in the past 2 decades. The compounds isolated from TwHF, including triptolide and tripdiolide, induce male infertility (Wang et al., 2014). Although the prospects of male antifertility drugs have been explored, further safety evaluation results of this drug are needed. Therefore, THH is likely to be a genotoxic agent. Chinese investigators are cautious and suggest that patients should avoid reproduction for 6 months after THH therapy (Liang et al., 2006). In addition, we emphasized the pharmacological effects of triptolide, celastrol and other important bioactive components, and great progress has been made in the identification of the mechanisms associated with these components, such as anti-inflammatory, immunosuppressive, and anti-tumour effects. At 50 nM, triptolide could completely block the LPS-induced PGE2 release. Further, triptolide at 50 or 100 nM in primary microglial cells was not toxic (Jiao et al., 2008). In addition, the animals pretreated with triptolide (100 mg/kg) show significant attenuation of LPS-induced proinflammatory cytokine release in serum, including TNF-a, IL-6, and IL-12, but not IL-1β (Huang et al., 2019). Triptolide could suppress CD80 and CD86 expressions on IFN-γ (500 kU/L) and LPS (1 mg/L)-activated THP-1 cells at 2.5–0.625 µg/L (Liu et al., 2005). Celastrol caused a dose- and time-dependent growth inhibition of A549 cells with an IC_50_ of 2.12 μM at 48 h treatment (Mou et al., 2011). Celastrol at the optimum concentration of 30 nM was able to protect the cells from mitochondrial dysfunction and insulin resistance (Abu Bakar et al., 2017).

At present, the studies *in vitro* and *in vivo* are relatively separate from each other. There is a lack of relevance between experiments *in vitro* and *in vivo* especially in the aspect of dosage. In future studies, more attention should be paid to research to understand whether the concentration used *in vitro* can achieve the same efficacy *in vivo*. In conclusion, some data suggest that triptolide may possess anti-angiogenic effect in RA both *in vivo* and *in vitro* assay systems by downregulating the angiogenic activators and inhibiting the activation of mitogen-activated protein kinase downstream signal pathway (Kong et al., 2013). Triptonide effectively inhibited pancreatic cancer cell-formed capillary-like structures *in vitro* and blood vessels *in vivo* through suppressing pancreatic cancer cell migration, invasion, and VM via inhibiting expression of tumour VM master gene VE-cadherin and pro-migratory gene chemokine C-X-C motif ligand 2 (CXCL2), mainly via reduction of gene promoter activity (Han et al., 2018). This is particularly crucial for the exploration of anti-tumour effect. However, this promising effect seems less to be displayed *in vivo*. It is to be due to the complex system of the body. Therefore, from the perspective of animals or even humans, we should construct a systematic experiment synchronously carried out the experiment *in vitro* and *in vivo*. Furthermore, the dosage for these studies should confirm the stability of the effect. In addition, researchers have discovered new pharmacological effects of these compounds, including alleviation of AD and insulin resistance as well as anti-obesity, and cardioprotective effects. However, these herbs also have potential multiorgan toxicity that has not been addressed for many years (Wang L. et al., 2018). Pharmacodynamic and toxicological studies of THH are closely related to pharmacokinetics and tissue distribution. Due to the lack of studies on human pharmacokinetics, it is unclear whether the pharmacological effects obtained at the concentrations of compounds used *in vitro* can achieve the same efficacy in humans. Therefore, various toxic reactions might occur in clinical application. Many studies have reported the metabolic processing of toxic active compounds *in vitro* and *in vivo*, aiming to reduce drug toxicity and improve the safety of these agents from a metabolic point of view or using different methods.

Alternatively, many low toxicity active analogues have been developed by drug synthesis researchers from natural active products of the genus *Tripterygium* (Shan et al., 2017; Ning et al., 2018). The Chinese pharmacopoeia indicates that THH patent medicine (Kunming Shanhaitang tablet) is made from extracts of THH dried roots, and this is a legally licenced drug in China. Kunming Shanhaitang tablet is commonly used to treat RA. Inhibition of IL-2 production by human peripheral blood lymphocytes through nuclear inhibition of transcriptional activation of NF-κB, prevention of T cell proliferation, induction of apoptotic death of T lymphocytes, reduction of PGE2 production in human monocytes and rheumatoid arthritis synovial fibroblasts, suppression of complement C3 and the expression of CD40 and B7 in activated human proximal tubular epithelial cells by triptolide indicates a wide range therapeutic target cells of this Chinese herb. Dex is a well-known classical glucocorticoid with potent anti-inflammatory and immunosuppressive effects when gave in pharmacological doses. Though the effect of triptolide is not so strong as Dex in the clinical managements, it shares the properties of Dex such as anti-inflammation and immunosuppression. Here, we demonstrated that DC is a target of triptolide. Due to DC has the unique property to activate native T cells and is required for the induction of a primary response, the suppression of DC function may very efficiently control the specific immune response. One of the mechanisms by which triptolide can suppress the immune response in humans is by inhibiting differentiation, terminal maturation and function of DC. In addition, triptolide abrogates the capacity of mature DCs to secrete IL-12 and also promotes DC apoptosis. These effects result in inhibition of alloreactive T cell activation. Suppression of DC may contribute to the actions of triptolide in the treatment of immune-related diseases and for the prevention of allograft rejection. Adjuvant treatment of facial corticosteroid addiction dermatitis with Kunming Shanhaitang tablet effectively improved symptoms and manifestations within 2 months and was more beneficial than topical drugs alone in significantly improving symptoms in the first 2 weeks. In addition, the effect was comparable to that of antihistamine combined with topical drugs or topical drugs alone (Xue and Wu, 2008). The injection of THH demonstrates a significant inhibitory effect on the production of haemolysin antibodies in mice, and the effect is more obvious with a long period of dosing (Li X, 2006). Tripterygium agents (TAs) extracted from TwHF are a complementary therapy used to treat eczema based on previous experience. However, TA has significant side effects in the treatment of specific eczema, but seems to be effective only in combination with some therapies. Thus, researchers believe that TA cannot be used clinically for eczema in general (Liu et al., 2019). Less toxic preparation of THH could potentially be used as an alternative. In previous separation and extraction experiments, most of the chemical active ingredients of THH were extracted with 95% ethanol, but it was found that column chromatography was replaced by sodium carbonate extraction for removing the acidic compounds and enriching epoxyditerpenoids and alkaloids in the extract. The therapeutic index (IC_50_/EC_50_) on murine macrophage Raw 264.7 cells and rat mesangial HBZY-1 cells of the extract prepared by sodium carbonate extraction was significantly higher than that of Tripterygium glycosides (0.8 and 5.2 vs. 0.3 and 2.6), while its cytotoxicity on human liver HL7702 cells was significantly lower (14.5 ± 1.4 vs. 6.8 ± 0.9,). Therefore, *Tripterygium* extract prepared by sodium carbonate extraction may represent a potentially optimal source of medicine with good therapeutic index (Fang et al., 2012). Therefore, many studies have shown that THH is a potential source of various drugs. Here, we consulted the literature and summarized the useful information and evidence. From the current research, it can be seen that a variety of active components of THH show significant efficacy and great potential in many major diseases. Toxicity is the main factor limiting THH in drug development. To date, the research on the mechanism of toxicity caused by THH is not sufficient. A full understanding of the mechanism of toxicity induced by THH is the premise to effectively reduce the toxicity of THH, and it is also the key to the development of THH efficacy. So do other plants of the genus *Tripterygium*. Through continuous in-depth studies of THH and TwHF, our understanding of their structure, pharmacology, toxicology and other aspects will be further advanced, which will provide a solid theoretical basis for their clinical application. In the future, if we focus on preserving the active ingredients and minimizing the toxic components of the preparations, these agents will certainly have broader prospects in various fields.

In conclusion, THH has abundant chemically active components, and some representative components (triptolide, celastrol, etc.,) have been demonstrated to have significant anti-inflammatory, immunosuppressive and anti-tumour effects. Meanwhile, the toxicity of THH cannot be underestimated. How to effectively control the toxicity of THH and obtain the maximum therapeutic effect is still a problem to be solved. The combination of traditional theory and modern advanced technology will be an important goal for the efficacy and safety of traditional Chinese medicine.

## Glossary

17β-HSD: 17β-hydroxysteroid dehydrogenase
2-HIB: 2-hydroxyisobutyrate
2-HIV: 2-hydroxyisovalerate
3β-HSD: 3β-hydroxysteroid dehydrogenase
ABCA1: ATP-binding cassette transporter A1
AD: Alzheimer’s disease
aGVHD: acute graft versus host disease
ALP: alkaline phosphatase
ALT: alanine aminotransfease
AML: acute myeloid leukaemia
AMPKα: Adenosine 5′-monophosphate (AMP)-activated protein kinase
APCs: antigen presenting cells
APL: acute promyelocytic leukemia
ARC: hypothalamic arcuate nucleus
**As**_**2**_ **O**_**3**_: arsenic trioxide
AST: aspartate aminotransferase
ATL: adult T-cell leukaemia/lymphoma
AUC: Area Under Curve
Bcl-xL: B-cell lymphoma-2
CD: Crohn’s disease
c-Fos: Cellular oncogene fos
CIA: collagen II-induced arthritis
c-Jun: Cellular Jun
c-Myc: cancer-Myc
COX: cyclo-oxygen-ase
CPT1A: Carnitine Palmitoyl transferase 1A
CRC: colorectal carcinoma
Ctr: calcitonin receptor
Ctsk: cathepsin K
CXCL2: C-X-C motif ligand 2
DCs: dendritic cells
Drp1: dynamin-related protein 1
EBV: Epstein-Barr virus
ERK: extracellular regulated protein kinases
Foxp3: Forkhead box P3
GA: glycyrrhetinic acid
GCAFs: gastric cancer-associated fibroblasts
GLUT1: glucose transporter 1
GLUT4: glucose transporter 4
GPx: glutathione peroxidase;
GSH: glutathione
HCV: hepatitis C virus
HIF-1α: hypoxia-inducible factor 1-α
HK2: hexokinase 2
HLA-DR: human leukocyte antigen DR
HO-1: haem oxygenase-1
HSF1: heatshocktranscription factor 1
HSP70: Heat shock 70 kDa protein
HUVEC: human umbilical vein endothelial cells
IFN-g: interferon gamma
IKK: IκB kinase
IL1R1: interleukin 1 receptor 1
IL-1β: interleukin-1β
IL-6: interleukin-6
iNOS: inducible Nitric Oxide Synthase (enzyme)
JNK1: c-Jun N-terminal kinase 1
LDH: Lactic Dehydrogenase
LDL/VLDL: low-density lipoprotein/very low-density lipoprotein
LPS: lipopolysaccharide
MAPK: mitogen-activated protein kinase
MCP: monocyte chemotactic protein
MDA: malondialdehyde
MDM2: murine double minute2
MDR: multidrug resistance
MIP: macrophage inflammatory protein
miR-96: microRNA-96
MLRs: mixed lymphocyte reactions
Mmp-9: matrix metalloprotein 9
MMPs: matrix metalloproteinases
MoDCs: monocyte-derived DCs
mTOR: mammalian target of Rapamycin
NAC: N-acetylcysteine
NADPH: nicotinamide adenine dinucleotide phosphate
NFATc: nuclear factor activated T-cells
NF-κB: nuclear factor kappa-B
NO: nitric oxide
OPG: osteoprotegerin
P450c17: cytochrome P450 17-hydroxylase
P450scc: P450 side-chain cleavage enzyme
PARP: poly ADP-ribose polymerase
PBMCs: peripheral blood mononuclear cells
PC: Pancreatic cancer
PFKP: Phosphofructokinase Platelet
PGE2: prostaglandin E2
P-gp: P-glycoprotein
PI3K: phosphatidylinositol 3-kinase
PKC: protein kinase C
PKCθ: Protein Kinase C θ
PLC: phospholipase C
proMMP-1: pro-matrix metalloproteinase-1
PTP: protein tyrosine phosphatase
RA: rheumatoid arthritis
RANKL: receptor activator of NF-κB ligand
RNS: reactive nitrogen species
ROS: reactive oxygen species
SOD: superoxide dismutase
StAR: steroidogenic acute regulatory protein
STAT: signal transducers and activators of transcription
TAB1: TAK1 binding protein
TAK1: transforming growth factor β-activated kinase 1
TAs: Tripterygium agents
TCM: Traditional Chinese medicine
TCPTP: T cell PTP
THH: Tripterygium hypoglaucum (Lévl.) Hutch.
TLR: Toll-like receptors
TNF-α: tumour necrosis factor-α
TRAIL: TNF-related apoptosis-inducing ligand
TRAP: tartrate-resistant acid phosphatase
Tregs: T regulatory cells
TwHF: Tripterygium wilfordii Hook F.
VEGF: vascular endothelial growth factor
XIAP: X-linked inhibitor of apoptosis protein
